# Peptide Model of the Mutant Proinsulin Syndrome. I. Design and Clinical Correlation

**DOI:** 10.3389/fendo.2022.821069

**Published:** 2022-03-01

**Authors:** Balamurugan Dhayalan, Michael D. Glidden, Alexander N. Zaykov, Yen-Shan Chen, Yanwu Yang, Nelson B. Phillips, Faramarz Ismail-Beigi, Mark A. Jarosinski, Richard D. DiMarchi, Michael A. Weiss

**Affiliations:** ^1^Department of Biochemistry and Molecular Biology, Indiana University School of Medicine, Indianapolis, IN, United States; ^2^Department of Biochemistry, Case Western Reserve University School of Medicine, Cleveland, OH, United States; ^3^Department of Physiology & Biophysics, Case Western Reserve University School of Medicine, Cleveland, OH, United States; ^4^Department of Medicine, Case Western Reserve University School of Medicine, Cleveland, OH, United States; ^5^Department of Chemistry, Indiana University, Bloomington, IN, United States

**Keywords:** monogenic diabetes, endoplasmic reticular stress, peptide chemistry, protein folding, oxidative folding intermediate

## Abstract

The mutant proinsulin syndrome is a monogenic cause of diabetes mellitus due to toxic misfolding of insulin’s biosynthetic precursor. Also designated *mutant INS-gene induced diabetes of the young* (MIDY), this syndrome defines molecular determinants of foldability in the endoplasmic reticulum (ER) of β-cells. Here, we describe a peptide model of a key proinsulin folding intermediate and variants containing representative clinical mutations; the latter perturb invariant core sites in native proinsulin (Leu^B15^→Pro, Leu^A16^→Pro, and Phe^B24^→Ser). The studies exploited a 49-residue single-chain synthetic precursor (designated DesDi), previously shown to optimize *in vitro* efficiency of disulfide pairing. Parent and variant peptides contain a single disulfide bridge (cystine B19-A20) to provide a model of proinsulin’s first oxidative folding intermediate. The peptides were characterized by circular dichroism and redox stability in relation to effects of the mutations on (a) *in vitro* foldability of the corresponding insulin analogs and (b) ER stress induced in cell culture on expression of the corresponding variant proinsulins. Striking correlations were observed between peptide biophysical properties, degree of ER stress and age of diabetes onset (neonatal or adolescent). Our findings suggest that age of onset reflects the extent to which nascent structure is destabilized in proinsulin’s putative folding nucleus. We envisage that such peptide models will enable high-resolution structural studies of key folding determinants and in turn permit molecular dissection of phenotype-genotype relationships in this monogenic diabetes syndrome. Our companion study (next article in this issue) employs two-dimensional heteronuclear NMR spectroscopy to define site-specific perturbations in the variant peptides.

## Introduction

**Figure 1:**
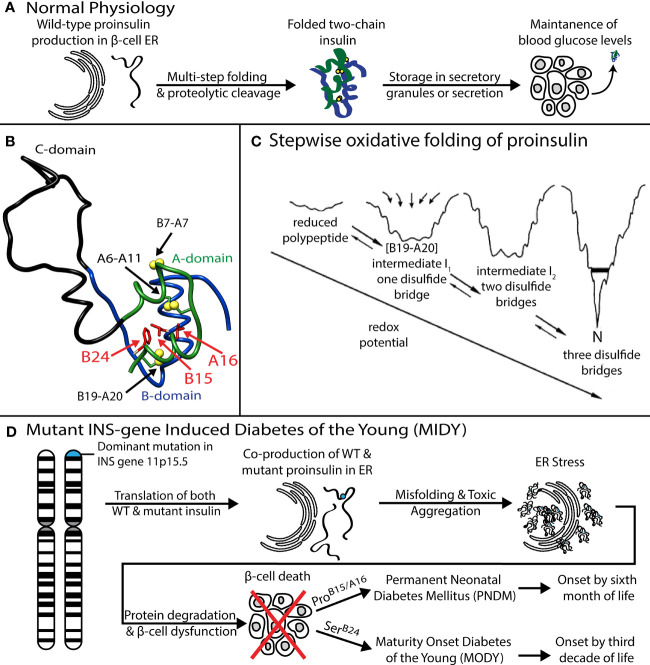
Genetic etiology, pathophysiology, and structural basis of Mutant Proinsulin Syndrome. **(A)** Overview of normal proinsulin production and maintenance of glucose homeostasis. **(B)** Solution structure of WT proinsulin (7) showing the A- (*green*), B- (*blue*), and C (*black*) domains and three native disulfide bridges (*yellow spheres*). Wild-type residues Phe^B24^, Leu^B15^ and Leu^A16^, which are examined in this study as sites of mutation, are shown as red side chains. **(C)** Folding of proinsulin proceeds *via* sequential disulfide linkage steps from the fully unfolded state through one (1SS), two (2SS), and three disulfide bonded (3SS) conformational ensembles before reaching the native state (N). Chemical trapping experiments showed that the formation of B19-A20 disulfide as the predominant key first step in the hierarchical disulfide pathway (8–10). Reprinted with permission from Hua QX, et al. Biochemistry. 2001;40:12299-311. Copyright (2001) American Chemical Society. **(D)** Dominant mutations in the *INS* gene causing co-production of both WT and mutant proinsulin, leading to ER stress, β-cell dysfunction, and, ultimately, diabetes. Clinically identified mutations Ser^B24^, Pro^B15^ and Pro^A16^ present with a spectrum of disease severity and age of diabetes onset.

The mutant proinsulin syndrome (MPS) is a monogenic cause of diabetes mellitus (DM) presenting at a broad range of ages: onset can occur either in the neonatal period, childhood, adolescence or early adulthood (1–4). Characterization of this syndrome and related mouse models (5, 6) has established the paradigm that DM may arise as a proteotoxic disorder of insulin biosynthesis (**Figure 1A**). Also designated *Mutant INS-gene Induced Diabetes of the Young* (MIDY) (11), MPS thus pertains to patients traditionally classified, on the basis of age at presentation, as either *Permanent Neonatal Diabetes Mellitus* (PNDM) or *Maturity Onset Diabetes of the Young* (MODY) (12). This phenotypic spectrum may reflect polygenic differences in β-cell biology (13) or intrinsic mutation-dependent biophysical properties of the variant proinsulins (11, 14, 15).

**Figure 2:**
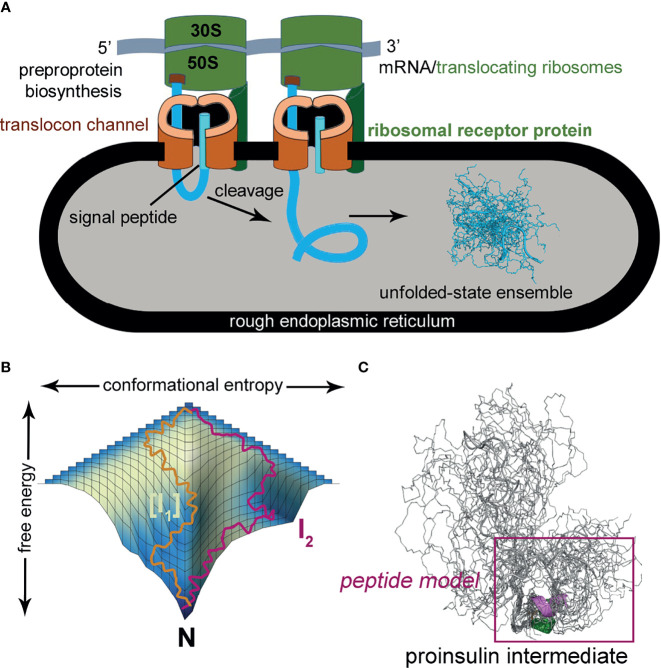
Peptide models of protein-folding intermediates probe critical biosynthetic steps. **(A)** Preproprotein biosynthesis in rough endoplasmic reticulum: nascent polypeptide chain (blue ribbon) enters ER (gray) with cleavage of signal peptide (cyan ribbon) *via* translocon channel (brown). Protein folding may in part be co-translational or begin from an unfolded-state ensemble (blue chains at right); this model was calculated by Xplor-NIH software and ensemble generated by PyMOL (ribbon and sticks). The cartoon was otherwise adapted from (56). **(B)** Energy folding landscape. Yellow and magenta lines indicate alternative paths to the native state (N): [I_1_], set of unobserved intermediate species; I_2_, observed intermediate that can accumulate in a kinetic trap. Panel adapted from reference (57). **(C)** Conformational ensemble of a one-disulfide proinsulin intermediate containing cystine B19-A20. A minimal model (magenta box) was designed based on a 49-residue peptide (50) as described in the main text. The present CD studies suggest formation of nascent α-helices in the central B domain and C-terminal A domain (dark green and magenta, respectively).

MIDY mutations are ordinarily dominant and associated with misfolding of the proinsulin variant in the endoplasmic reticulum (ER), leading to β-cell dysfunction and eventual death [(16); for reviews, see (17, 18)]. Whereas the majority of MIDY mutations introduce or remove a Cys residue—in either case leading to an odd number of thiol groups and hence risk of aberrant intermolecular disulfide pairing (19)—PNDM- and MODY phenotypes are also associated with non-cysteine-related mutations (20). The latter amino-acid substitutions generally occur at sites conserved among vertebrate insulins [and in most cases also shared by vertebrate insulin-like growth factors (IGFs) (21)]. These mutational “hot spots” define the structural framework of native insulin (**Figure 1B**) and are of mechanistic interest as putative molecular determinants of protein folding efficiency (20, 22, 23). The present study, based on the oxidative refolding pathway of native proinsulin (**Figure 1C**), describes the design and chemical synthesis of a single-chain peptide model of a key on-pathway proinsulin folding intermediate (8, 24). This model enables comparative biophysical studies of representative MIDY “hot-spot” mutations with neonatal or delayed disease onset (**Figure 1D**). First introduced in studies of bovine pancreatic trypsin inhibitor (25), peptide models of protein-folding intermediates have provided a general approach toward dissecting critical molecular interactions guiding the conformational search of a nascent polypeptide (see **Box 1** and **Figure 2**) (31, 32).

Box 1 **Peptide models of protein-folding intermediates.**

Studies of the mutant proinsulin syndrome (17) have built on general principles of cell biology and protein chemistry established over the past sixty years (26–28). This deep interdisciplinary background highlights mechanisms underlying the biosynthesis of disulfide-stabilized secretory proteins from the scale of organelles and macromolecular complexes (29, 30) to the molecular biophysics of a nascent polypeptide chain’s conformational search (31–34).

Ribosomal translation at the outer surface of the rough ER is coupled to cleavage of the signal peptide and associated translocation into the ER [**Figure 2A**; for review, see (37)]; the latter environment provides chaperones and oxidative machinery for disulfide bond formation, rearrangement, and quality control (38–42). These general processes pertain to β-cell physiology and dysfunction in DM (17, 35, 36). Although initial steps of protein folding can in some cases be co-translational (30), the nascent proinsulin chain is likely to form an unfolded-state ensemble (at right in **Figure 2A**) to enable initial pairing of two cysteines distant in the sequence (Cys^B19^ and Cys^A20^; residues 43 and 109 in preproinsulin) (9, 24, 43). The present study has exploited a subset of diabetes-associated mutations to investigate such long-range pairing.

Analysis of atomic-scale events in the *in vitro* refolding of polypeptide chains and their computational simulation (44, 45) provide insight into the challenges faced in cellular protein biosynthesis (27, 46). Folding is visualized as proceeding through funnel-shaped free-energy landscapes (**Figure 2B**), in general *via* multiple trajectories (*e.g.*, yellow or magenta lines) (31). Dissection of globular proteins into peptide models, pioneered by Oas and Kim in 1988 (25) has been broadly influential in enabling key steps to be identified (47). Applications have been described to both oxidative folding intermediates (25), the rapid autonomous folding of disulfide-free subdomains (48) and fragments containing engineered disulfide bridges (49). Use of peptide models may circumvent the usual cooperativity of globular protein folding, which can obscure discrete steps (25). The latter perspective has been reinforced by studies of intact proteins by native-state amide-proton exchange kinetics (34).

The present studies have exploited a peptide model of a key one-disulfide proinsulin folding intermediate (simulated ensemble in **Figure 2C**); its features favor formation of on-pathway nascent structure [see also **Figure 9** and Discussion (50)]. Peptide design builds on prior studies of insulin-related polypeptides lacking specific disulfide bridges (10, 51–55). To our knowledge, this is the first investigation of clinical mutations in a peptide model of a proinsulin folding intermediate.

**Figure 3:**
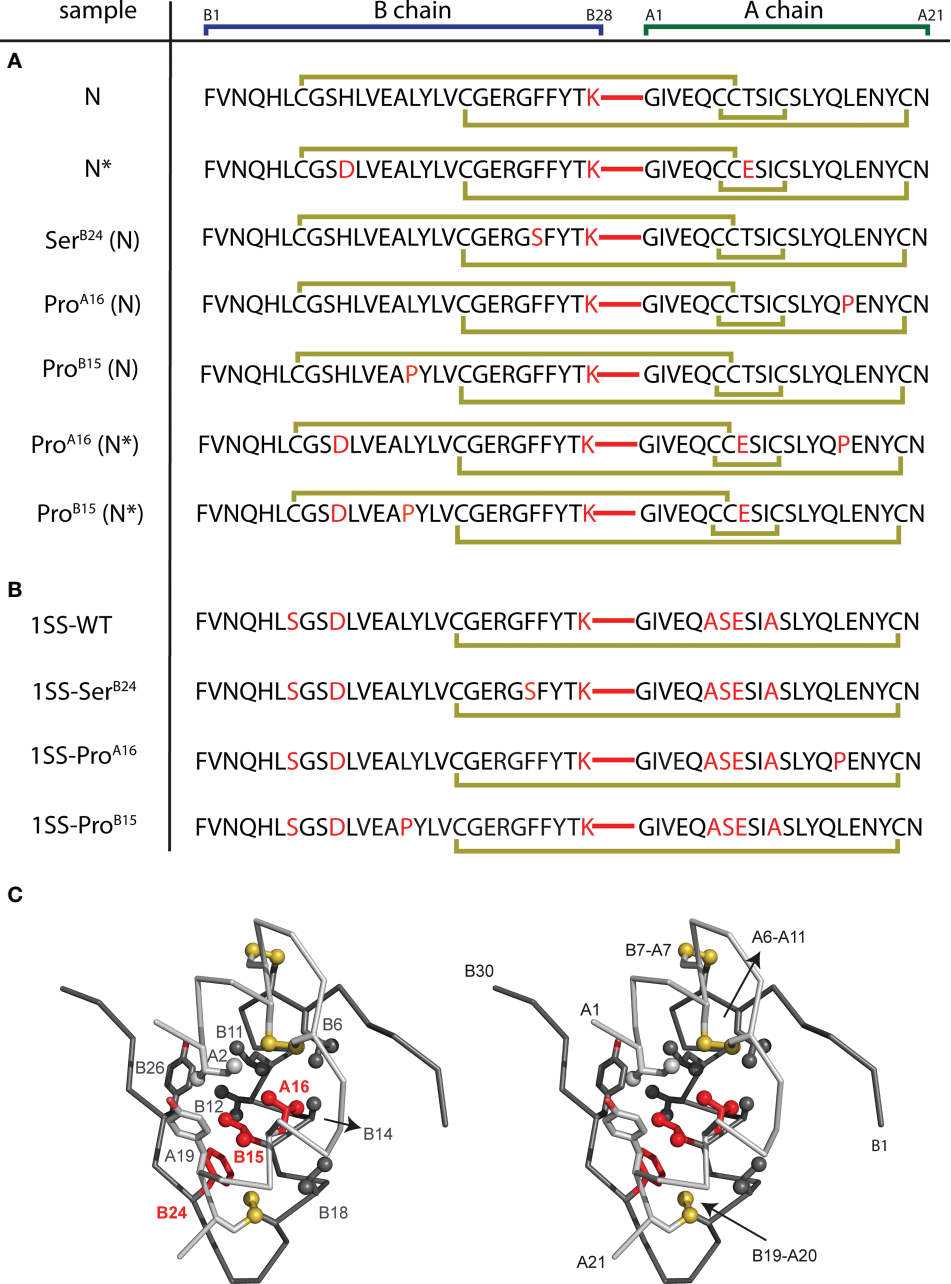
DesDi variant protein sequences. **(A)** Sample names and sequences of three disulfide bond containing analogs (N and N*). **(B)** Sample names and sequences of one disulfide-bond-containing analogs ([B19-A20]; abbreviated as 1SS). Yellow lines show disulfide linkages. A red line connecting the C-terminal B-domain and N-terminal A-domain sequences signifies the presence of a peptide bond between residues B28 and A1. All 1SS samples and N* have additional mutations Glu^A8^ and Asp^B10^ to enhance solubility at neutral pH (e.g., to enable the high protein concentrations needed for NMR spectroscopy). Residues in *red* highlight mutations to the WT insulin sequence. **(C)** Stereo view showing ribbon model of an insulin monomer [as extracted from the T_6_ Zn hexamer; PDB entry 4INS (60)]. Sites of clinical mutation investigated here [B15, B24 and A16; highlighted in red] and neighboring residues are shown as sticks. Sulfur atoms in disulfide bridges are shown as gold spheres, and selected side-chain methyl groups as spheres (one-third van der Waals radius). A- and B chains are otherwise shown in light and dark gray, respectively.

The present peptide model contains a single disulfide bridge, internal cystine B19-A20, the first partial fold to accumulate in chemical-trapping studies of proinsulin (or homologous IGF-I) refolding *in vitro* (8, 9, 24) (see Supplemental Discussion regarding effects of pH in such refolding assays). Its framework derives from a foreshortened single-chain synthetic precursor of insulin optimized for efficiency of disulfide pairing (50). Designated “DesDi”, the parent analog comprises 49 residues (**Figure 3A**): B-chain residues B1-B28 followed by A-chain residues A1-A21. Substitution of Pro^B28^ by Lys enables enzymatic cleavage to liberate an active two-chain insulin analog (50). Our one-disulfide model contains pairwise substitution of cystine B7-A7 by Ser and pairwise substitution of cystine A6-A11 by Ala (**Figure 3B**). Segmental α-helical propensity and solubility were augmented by substitutions His^B10^→Asp (native α-helix α_1_ spanning residues B9-B19) and Thr^A8^→Glu (native α-helix α_2_; A1-A8) (58, 59). Representative MIDY mutations (Leu^B15^→Pro, Leu^A16^→Pro, and Phe^B24^→Ser) were introduced into the parent DesDi framework with six cysteines (“native state”) and the peptide model (“1SS”). These amino-acid substitutions were chosen based on phenotype and structural interest: the proline variants (each neonatal in onset) are predicted to perturb nascent α-helical folding and native packing of the hydrophobic core (20, 60) whereas Ser^B24^ [with onset in adolescence or early adulthood (61)] perturbs the “aromatic anchor” of the B-chain β-strand (B24-B28) (60, 62, 63). Each of these conserved side chains contributes to core packing near internal cystine B19-A20 (**Figure 3C**). A related two-chain one-disulfide model of the homologous IGF-I folding nucleus has previously been described (51); the corresponding three side chains (IGF-I residues Leu14, Phe23 and Leu57) were observed to participate in its molten native-like structure at low temperatures.

The present study (the first of two in this issue) describes the synthesis and respective foldabilities of the above set of native state and 1SS 49-residue peptides. Circular dichroism (CD) is employed to probe α-helix contents, thermal unfolding profiles and thermodynamic stabilities. Characterization of such chemical and biophysical properties were extended through cell-biological assays of ER stress induced in human cells on transient expression of the corresponding mutant proinsulins. Striking correlations were observed among foldability, nascent α-helical folding and extent of ER stress in human cell culture. Our companion study further interrogates the nascent structures of these peptide models and their mutational perturbation by ^1^H and ^1^H-^13^C NMR spectroscopy [(64); following article in this issue]. Together, our findings establish a general platform for biophysical studies of a subset of MIDY mutations in relation to molecular mechanisms of proinsulin biosynthesis in pancreatic β-cells.

## Materials and Methods

### Automated DesDi Peptide Synthesis

Peptides were synthesized either with an ABI 433A Peptide Synthesizer (Applied Biosystems) or Tribute 2-Channel peptide synthesizer (Gyros Protein technologies) using a preprogrammed solid-phase fluorenylmethyloxycarbonyl (Fmoc) protocol designed for standard 0.1 mmol scale syntheses. ABI protocols consist of the following modules: cycle-1 [d], cycle-2 [aibde], cycle-3 with number of repetitions equal #aa-1 [afgbde], cycle“#aa+2” [ffbdc], where #aa is a number of amino acids in the sequence. Automated couplings utilized diisopropylcarbodiimide (DIC)/6-Cl-hydroxybenzotriazole (6-Cl-HOBt) in *N*-Methyl pyrrolidone (NMP) whereas Fmoc deprotections used 20% piperidine in NMP. α-carboxyl-protected Asp was used in place of Asn in all syntheses of DesDi analogs to accommodate the use of ChemMatrix^®^ Rink-Amide resin (loading = 0.46 mmol/g). The Tribute peptide synthesizer used heating protocols: couplings were done for 6 min at 60°C except for Cys/His (2 min at 25°C, then 5 min at 60°C) and Arg (20 min at 25°C, then 5 min at 60°C); deprotection was done twice (30 sec at 50°C, then 3 min at 50°C). Reagent conditions were otherwise similar to ABI protocols except that DMF was used as solvent and choice of the resin was H-Asn(Trt)-HMPB-ChemMatrix^®^ resin. Peptides were cleaved with TFA cocktail (2.5% vol/vol of each: β-mercaptoethanol, triisopropylsilane, anisole, and water) followed by ether precipitation.

### Folding and Purification of N and N* DesDi Analogs

Crude peptides from ether precipitation were dissolved in glycine buffer (20 mM glycine and 2 mM cysteine hydrochloride, pH 10.5) to a final peptide concentration of 0.1 mM. The pH of this solution was readjusted to 10.5 to account for traces of residual TFA present in lyophilized peptides. This solution was then stirred while open to air at 4°C until reaction completion (usually overnight). After monitoring of the folding reaction by analytical HPLC to determine the extent of conversion, the pH of the solution was then lowered to ~2.0 with 5N HCl to neutralize the folding reaction. Folded peptide was then purified by preparative rp-HPLC using Waters 2545 Quaternary pumping system equipped with FlexInject. Chromatographic separations were performed on a C4 Proto (20x250 mm) 300 Å, 10 μm, Higgins Analytical Inc. column, using 25-50% solvent B (0.1% TFA in acetonitrile) in solvent A (0.1% TFA in H_2_O) over 35 minutes (min) at a flow rate of 20 ml/min with detection by UV absorption at 215 nm. Fractions containing clean peptide were pooled and lyophilized. Purity of the materials was confirmed by LCQ Advantage Ion Trap Mass Spectrometer System coupled to an Agilent 1100 Series HPLC system. Masses were obtained by online electrospray mass spectrometry. MS data shown were collected across the entire principal UV-absorbing peak in each chromatogram; LC-MS retention times and mass verification are given in **Table S1**.

### Folding and Purification of [B19-A20]-SS DesDi Analogs

Purification of single-disulfide analogs was performed in a two-step process. First, the crude peptide was fully dissolved in pH 11 buffer in presence of excess DTT. The pH was then lowered to 8.0 before purification by semi-preparative rp-HPLC under alkaline conditions using 25 mM ammonium bicarbonate buffer (pH 8.0) and acetonitrile (60 min gradient of 20→50%) as eluents on a TRIART C18, 250 x 10mm 5μm, 120Å column. Fractions containing linear, fully reduced peptides were pooled and lyophilized. Folding was performed either using room air oxidation as described for N and N* analogs or utilizing cysteine-cystine redox pair. For the latter, peptides were dissolved in 20 mM glycine buffer (pH 10.5) at a final 0.1 mM peptide concentration followed by addition of 1:1 cystine/cysteine (2 mM each). Folding was allowed to proceed for 1 hour (hr), followed by purification by rp-HPLC using the same 20→50% acetonitrile elution gradient as described above. Collected fractions were pooled and lyophilized. When necessary, an additional purification using acidic RP-HPLC conditions was performed. Peptide purity was confirmed by analytical LC-MS as described for N and N* DesDi analogs above (LC-MS retention times and mass measurements are given in **Table S1**).

### Two-Chain DesDi Conversion

In cases of three disulfide containing DesDi analogs, single-chain insulins were converted to two-chain version by Lys-specific enzyme. In a typical experiment, single-chain DesDi analog was treated with Endo Lys-C enzyme (65) in 25 mM Tris base, 100 mM urea buffer (pH 8.5) at 12°C water bath for 24 h. After analytical rp-HPLC indicated two-chain conversion (typically 60-80%), the reaction mixture was acidified and purified on semi-preparative rp-HPLC. Fractions containing clean protein were pooled, lyophilized and masses were confirmed by LC-MS.

### Purification of Insulin Analogs

Wild-type human insulin and insulin *lispro* were purified from U-100 pharmaceutical formulations of Humulin^®^ and Humalog^®^ (Eli Lilly and Co.), respectively, using preparative rp-HPLC (C4 10μm 250×20mm Proto 300 Column; Higgins Analytical, Inc.) utilizing Buffer A (0.1% TFA in H_2_O) and a 10-min elution gradient of 20→70% Buffer B (0.1% TFA in acetonitrile). Following lyophilization of the collected protein fraction, purity was verified using analytical rp-HPLC (C4 5μm 250×4.6mm Proto 300 Column; Higgins Analytical, Inc.) with a 35-min elution gradient of 25→50% Buffer B; molar mass was verified with an Applied Biosystems 4700 Proteomics Analyzer utilizing MALDI-TOF in reflector mode; chromatographic retention times and mass measurements for these clinical analogs are given in **Table S1**.

### Nuclear Magnetic Resonance (NMR) Spectroscopy

All spectra were acquired at a protein concentration of ~0.2 mM in 100% D_2_O (pD 7.4) at 35°C with a Bruker AVANCE 700-MHz spectrometer, as described (23). All chemical shifts were calibrated in parts per million (ppm) relative to 4,4-dimethyl-4-silapentane-1-sulfonic acid (DSS) as an internal standard. The spectrometer was equipped with ^1^H, ^19^F, ^13^C, ^15^N quadruple resonance cryoprobe.

### CD Spectropolarimetry

**Figure 4:**
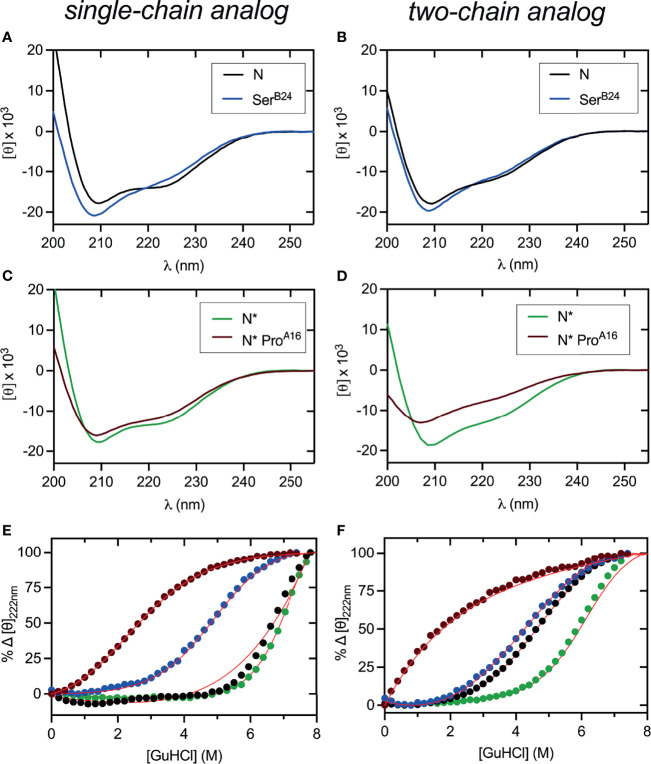
Far UV-CD and stability studies of three disulfide containing DesDi variants. **(A, B)** CD wavelength scans of samples (N) acquired at 25°C. **(C, D)** CD wavelength scans of N*-samples acquired at 25°C. In panels **(A–D)**, left hand side represent single-chain data and right-hand side two-chain data. **(E, F)** Comparative CD-guanidine titrations of all three disulfide samples (solid lines are fits). Color legend: N (black), N* (green), Ser^B24^ (blue) and N* Pro^A16^ (maroon). Δ*G*_u_ values obtained by titration curve fitting are presented in **Table 1**. All titrations were performed at 25°C, except for N (37°C), Ser^B24^ (37°C) and N* (50°C) single-chain samples where higher temperature was used to enhance their unfolding transition.

Far ultraviolet (255-190 nm) CD spectra were obtained at high signal-to-noise for WT insulin, insulin *lispro*, and all [B19-A20], N and N* DesDi peptides (summarized in **Figure 4**) using a CD spectropolarimeter (Aviv-400 or Jasco J-1500) equipped with temperature control and an automated titration unit. Samples were prepared at a concentration of 20-70 μM protein in degassed potassium phosphate (10 mM KH_2_PO_4_/K_2_HPO_4_ with 50 mM KCl), brought to pH 7.4 with KOH, and placed in a parafilm-sealed 1-mm pathlength quartz cuvette. Automated macros were utilized that acquired full far-UV CD spectra (255-190 nm) in 2°C steps from 4°C to 40°C (plus 25°C and 37°C) with a wavelength resolution of 0.5 nm and 30 sec. photocount averaging time. Following this, spectra were acquired from 4-88°C in 4°C steps. To reduce acquisition time at high temperatures, ellipticity measurements made above 40°C included only wavelength sets of 254(± 1), 222(± 1) and 208(± 1) nm using a 0.5-nm wavelength resolution and 30-sec. detector averaging time. Buffer-only CD spectra were obtained using degassed buffer with no protein at temperatures of 4, 25 and 37°C using a 0.5-nm wavelength resolution and 90 sec. averaging time. The linear temperature dependence at all wavelengths of buffer-only spectra allowed interpolation and extrapolation of buffer-only spectra at any temperature. Extrapolated reference spectra were then subtracted from all CD spectra acquired at the same temperature. Estimates of secondary-structure content were obtained from normalized spectra acquired at 4, 25 and 37°C using the SELCON-3 algorithm packaged with the CDPro spectral analysis software (66–68).

The temperature dependence of protein folds was assessed by plotting the average of molar ellipticity values at wavelengths straddling the α-helix sensitive wavelength of 222 nm (i.e., 221-223 nm with 0.5-nm resolution) against temperature. The quantity resulting from averaging wavelengths 221-223 nm, $\langle {\left[\theta \right]}_{222\pm 1nm}\rangle$ , enhances signal-to-noise while ensuring that any difference in ellipticity that may be observed between sequential temperature steps is not confounded by random error.

### Quantitation of Samples and Normalization of CD Spectra

CD samples were quantitated *via* reference-subtracted UV-Vis spectra acquired in 10 mM potassium phosphate (pH 7.4) with a UV-Vis spectrometer (Aviv Biomedical Inc., Lakewood, NJ) and a 3-mm quartz cuvette. Protein concentrations in potassium phosphate, [*C*]*_KPi_*, were calculated using absorbance at λ=280 nm and estimated extinction coefficients predicted by the online ExPASy ProtParam tool (69), which estimates molar absorptivity using the amino-acid sequence and cysteine sulfur oxidation states of each peptide. Calibration of these estimated extinction coefficients, which do not anticipate the effects of intramolecular dipole-dipole interactions in a folded protein, was achieved by quantifying [*C*]*_GuCl_*, the concentration of the same amount of protein in a buffer containing 8M guanidine hydrochloride, 10 mM potassium phosphate (pH 7.4) and 50 mM KCl; and then calculating a unique correction factor *M_G_* = [*C*]*_KPi_*/[*C*]*_GuCl_* for each sample. As DesDi protein samples containing all three native disulfide bridges do not fully unfold in 8M guanidine, extinction coefficients for N, N* and WT insulin were calibrated by determining amino-acid compositions of the samples from three cycles of N-terminal Edman degradation sequencing using an Applied Biosystems Procise 494 Sequencer. This approach was validated by the fact that calibration *via* UV-Vis spectroscopy with potassium phosphate buffer containing guanidine hydrochloride yielded the same correction factors for wildtype insulin. Percent error in the estimated extinction coefficient of WT insulin and insulin *lispro* was ~4% whereas that of N* and N was 9% and 24%, respectively. The predicted extinction coefficients of all 1SS peptides, which are mostly unfolded in zero denaturant (see **Figure 4**), had <0.5% deviation from the experimental value. CD spectra reporting molar ellipticity per residue, [Θ], were calculated by dividing raw ellipticities by the corrected protein concentration ([*C*]*_cal_* = [*C*]*_KPi_*/*M_G_*) and the number of amino acids, *N*, for each protein.

### CD-Monitored Guanidine-Induced Unfolding Studies

Thermodynamic stabilities of all insulin peptides in 10 mM potassium phosphate buffer (pH 7.4) at 25°C were determined using guanidine hydrochloride titrations monitored by CD at the α-helix-sensitive wavelength 222 nm as described (23). Using non-linear least squares regression, plots of ellipticity vs. guanidine concentration were fit to a two-state unfolding model (70):


[1] $$\Theta (c)=\frac{\Theta_{A}+\Theta_{B}e^{(-\Delta G-mc)/RT}}{1+e^{(-\Delta G-mc)/RT}}.$$

where Δ*G* is the Gibbs free energy of unfolding, *C* is guanidine concentration, *R* is the ideal gas law constant, *T* is temperature, and Θ*_A_* and Θ*_B_* are baseline ellipticity values reflecting the folded and unfolded state. Baseline ellipticities were calculated *via* simultaneous fitting of linear equations Θ*_A_*(*c*) = Θ*_A_* + *m_A_c* and Θ*_B_*(*c*) = Θ*_B_* + *m_B_c* as described (71).

### GSH-GSSH Assay

1SS-peptides (50 μg, 45 μM final concentration) were treated with 25 mM reduced glutathione (GSH) and 5 mM oxidized glutathione (GSSH) in 200 μL buffer (20 mM sodium phosphate [pH 8.2] and 100 mM NaCl) and allowed to attain equilibrium at 25°C. After 3 hrs, an aliquot was acidified with 6 M guanidine hydrochloride in 0.1% trifluoroacetic acid and analyzed by analytical rp-HPLC using 5-65% solvent B (0.1% TFA in acetonitrile) in solvent A (0.1% TFA in water) over 23 min.

### Proinsulin Constructs

Plasmids expressing full-length human proinsulin or variants were constructed by polymerase chain reaction (PCR). Mutations in proinsulin were introduced using QuikChange™ (Stratagene). Constructions were verified by DNA sequencing.

### Mammalian Cell Culture and ER Stress Assays

Human embryonic kidney 293T cells were purchased from American Type Culture Collection and cultured in Dulbecco’s Modified Eagle Medium (DMEM), supplemented with 10% fetal bovine serum (FBS), 1% penicillin/streptomycin as recommended. Transfections were performed using Lipofectamine 3000 as described by the vendor (Invitrogen). Transfected HEK 293T cells were subjected to the Bio-Rad one-step real-time qPCR protocol. Readouts were provided by the up-regulation of ER stress markers CHOP and BiP. The gene expression values were normalized by the expression of the gene encoding glyceraldehyde 3-phosphate dehydrogenase (GAPDH) as internal control. The mRNA abundances were measured in triplicate. In Western blot assay probing ER stress markers (72, 73), after 24 hr post transient transfection, cells were lysed by RIPA buffer (Cell Signaling Technology; CST). Protein concentrations in lysates were measured by BCA assay (Thermo) and subjected to 4-20% SDS-PAGE and WB using anti-pPERK. Anti-PERK, anti-BiP and anti-CHOP antibodies (CST) at a dilution ratio of 1:1000; GAPDH provided a loading control.

### Rat Experiments

Animals were maintained in accredited facility of Case Western Reserve University School of Medicine. All procedures were approved by the Institutional Animal Care and Use Committee (IACUC) office of the University. Animal care and use was monitored by the University’s Veterinary Services.

### Measurement of the Glucose-Lowering Effect of Insulins in Diabetic Rats

Male Lewis rats (average body mass of ~300 g) were rendered diabetic by streptozotocin (STZ) as described (23). Insulin analogs were dissolved in Lilly^®^ Diluent buffer with the specified dose and injected in 100 µL/300 g rat. Lispro insulin (KP) was diluted as appropriate in Lilly^®^ Diluent buffer. Control rats received the appropriate volume of the Lilly buffer. For intravenous (IV) injection, rats were anesthetized in a chamber for 5 min using a mixture of 5% isoflurane and 95% oxygen. Following cleaning of the tail, rats were injected while under anesthesia using the lateral tail vein. For subcutaneous (SQ) experiments, rats were injected under the skin into the soft tissue in the posterior aspect of the neck. Following injection, blood glucose was measured using a small drop of blood obtained from the clipped tip of the rat’s tail using a clinical glucometer (EasyMax^®^ V Glucose Meter, Oak Tree Health, Las Vegas, NV). Blood-glucose concentrations were measured at time t=0, and every 10 min for the first hr, every 20 min for the second hr, every 30 min for the third hr, and then each hr for the rest of the experiment.

### Molecular Modeling

Structural ensembles were calculated by simulated annealing using *XPLOR-NIH* (74–76). A model of the one-disulfide proinsulin intermediate (containing cystine B19-A20; see **Box 1**) was generated using distance restraints pertaining to residues A16-A21 and B15-B26 as observed in an engineered proinsulin monomer (7). A similar modeling protocol was employed to generate ensembles for 1SS-DesDi variants; selected distance restraints were extracted from NOESY spectrum of an engineered insulin monomer (23). For the parent 1SS DesDi model, helix-related distance restraints were corroborated by NMR [see companion study (64)]. To allow for protein flexibility in these partial folds, upper bounds on long-range distance restraints were increased by 3 Å relative to NMR-derived bounds obtained in prior studies of insulin and proinsulin (7, 23).

## Results

Eleven 49-residue peptides were prepared by solid-phase peptide chemistry (**Figure 3**); the red segments represent a peptide bond between residues B28 and A1. Seven peptides contained insulin’s canonical six cysteines with intended disulfide pairing indicated in gold (**Figure 3A**). As expected (50), peptide N and N* (DesDi and [Asp^B10^, Glu^A8^]-DesDi, respectively) underwent oxidative folding with high efficiency to yield a single predominant product (**Table 1**). DesDi’s foldability was decreased or blocked by the MIDY substitutions in order Ser^B24^ (least perturbed relative to the N parent peptide) >> Pro^A16^ = Pro^B15^ (no folded product detected). Introduction of N* substitutions Asp^B10^ and Glu^A8^ rescued inefficient but detectable folding of Pro^A16^-N* but not Pro^B15^-N* (**Table 1**). Four 1SS model peptides were also synthesized (**Figure 3B**). Because these contain only two cysteines, disulfide pairing was efficient in each case, including in the presence of Pro^A16^ and Pro^B15^. Reverse-phase HPLC retention times and molecular masses are given in **Table S1**. Two-chain versions of N/N* analogs and the parent 1SS model peptide were obtained following enzymatic cleavage with Lys-C protease (50). Analytical rp-HPLC chromatograms and LC-MS profiles are provided as **Figures S1–S15**.

**Table 1:** Folding yields of DesDi synthetic precursors and thermodynamic stabilities.

native state peptide domain	isolated yield (mg)^a^	Δ*G*_u_(kcal/mol)^b^	*m* value(kcal/mol/M)	C_mid_(M)
single-chain (N)^c^	21.7 mg	>4^c^	N.D.^c^	N.D.^c^
single-chain (N*)^c^	21.2 mg	7.6 ± 0.4^c^	0.99 ± 0.02	7.68 ± 0.1
Ser^B24^ single-chain (N)^c^	12.0 mg	3.4 ± 0.1^c^	0.69 ± 0.02	4.93 ± 0.1
Pro^A16^ single-chain (N)^d^	0	–	–	–
Pro^A16^ single-chain (N*)^d^	2.1 mg	0.9 ± 0.1	0.51 ± 0.02	1.76 ± 0.1
Pro^B15^ single-chain (N)^e^	0	–	–	–
Pro^B15^ single-chain (N*)^e^	0	–	–	–

a Isolated yield represents amounts after rp-HPLC purification from a folding reaction of 100 mg reduced polypeptide.

b ΔG_u_ values provided were obtained from curve fitting of CD-guanidine titrations to a two-state unfolding transition model as described in Methods (23).

c CD-guanidine titrations performed at 37°C for N and 50°C N* (see **Figure 4**) yielded partially folded peptides at the maximum guanidine concentration. Fitting of the N* titration was successful (R^2 =^ 0.9994), but analysis of the N titration curve could only place a lower limit on thermodynamic stability of 4 kcal/mol.

d No product obtained. Folding was nonetheless rescued in part by Asp^B10^ and Glu^A8^ substitutions (N*).

e No isolable product obtained. In the case of Pro^B15^, inclusion of stabilizing substitutions Asp^B10^ and Glu^A8^ (N*) did not rescue foldability: only disulfide isomers were observed.

CD studies were conducted of the native-state analogs as single chains (left-hand panels in **Figure 4**) and on cleavage of the Lys^B28^-Gly^A1^ peptide bond (right-hand panels in **Figure 4**).^1^ MIDY mutations Ser^B24^ and Pro^A16^ are each associated with reduced α-helix content (**Table 2**).^2^ The extent of perturbation was more marked in the case of Pro^A16^, especially in the two-chain context (**Figure 4D** and **Table 2**, row 8 *versus* row 10). Thermodynamic stabilities were inferred from CD-detected guanidine denaturation studies (**Figures 4E, F**). Application of a two-state model provided estimates of free energies of unfolding (Δ*G*_u_; **Table S1**, column 3). In accordance with their relative susceptibilities to guanidine-induced unfolding, apparent by qualitative inspection of the denaturation data, Pro^A16^ is more profoundly destabilizing than is Ser^B24^. In each case imposition of the B28-A1 peptide bond enhances stability [which may rationalize its utility as a vehicle for oxidative folding (50)]. The two-chain Pro^A16^ N* analog did not exhibit a cooperative unfolding transition, and so its stability could not be estimated by this method. The stabilities of corresponding Pro^B15^ native state analogs could not be assessed due to absence of folded product. Functional studies of two-chain versions of Ser^B24^-N demonstrated reduced but substantial activity in a rodent model of DM (**Figure S16**) in accordance with past studies (23, 61, 78). The two-chain derivative of Pro^A16^-N* was inactive as was the two-chain derivative of the 1SS parent peptide.

**Table 2:** CD-derived secondary-structure contents^a^.

sample Name	α-helix	β-sheet	disordered
***A)***			
insulin *lispro*	38 – 40%	9 – 10%	32 – 33%
wild-type insulin^b^	47 – 56%	3 – 10%	24 – 29%
single-chain (N)	49 – 64%	2 – 10%	24 – 27%
single-chain (N*)	49 – 58%	4 – 10%	24 – 26%
Ser^B24^ single-chain (N)	55 – 61%	3 – 7%	27 – 28%
Pro^A16^ single-chain (N*)	43 – 48%	11 – 14%	27 – 28%
two-chain DesDi (N)	47 – 53%	10 – 12%	26 – 28%
two-chain DesDi (N*)	56 – 61%	7 – 10%	24 – 26%
two-chain Ser^B24^ (N)	50 – 58%	5 – 6%	27 – 29%
two-chain Pro^A16^ (N*)	25 – 26%	22 – 23%	31 – 32%
***B)***			
1SS-WT	27 – 30%	18 – 20%	30 – 31%
1SS-Ser^B24^	19 – 20%	26 – 27%	31 – 32%
1SS-Pro^A16^	12 – 14%	32 – 33%	30 – 31%
1SS-Pro^B15^	10 – 11%	29 – 32%	32 – 34%

a Total percent α-helix, β-sheet, and disordered coil were obtained from spectra acquired at discreet temperatures of 4°C, 25°C, and 37°C using the SELCON-3 algorithm (66–68). Estimated percentages are presented as the minimum to maximum content calculated across the three sampled temperatures.

b Estimates were calculated from WT insulin spectra to confirm that SELCON-3 processing of our own CD data gives values that match those published in the literature (77).

**Figure 5:**
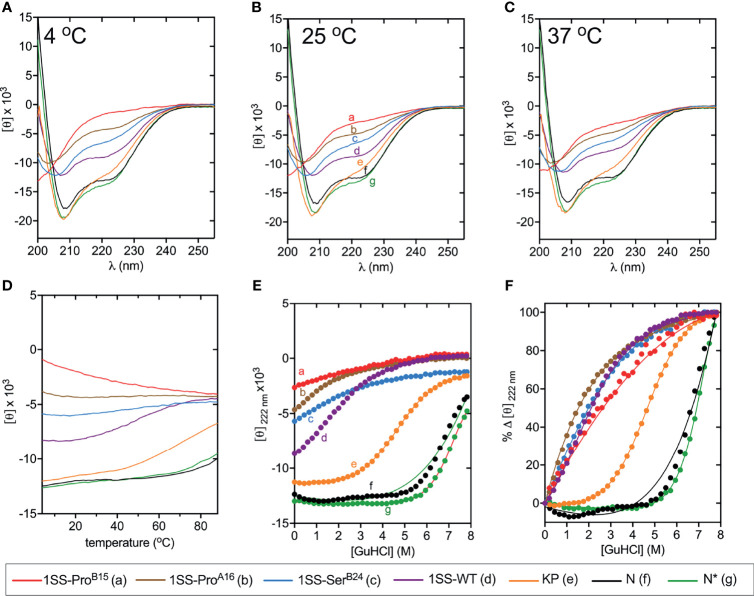
Folding and thermodynamic stability of 1SS variants and controls. **(A–C)** CD wavelength scans of all 1SS variants, N, and N* acquired at **(A)** 4°C, **(B)** 25°C, and **(C)** 37°C. The weak temperature dependence of each secondary structure is visualized with **(D)** a plot of 〈[θ]_222±1nm_〉 versus temperature. **(E, F)** Comparative CD-guanidine titrations of insulin *lispro* and all 1SS samples (solid lines are fits). Color legend: insulin lispro (orange), N* (green), N (black), 1SS-WT (purple), 1SS-Ser^B24^ (blue), 1SS-Pro^A16^ (brown) and 1SS-Pro^B15^ (red). ΔG_u_ values obtained by titration curve fitting are presented in **Table 1** and **Table S1**. To enhance their unfolding transition, N and N* titrations were performed at 37°C and 50°C, respectively; all other titrations were performed at 25°C.

CD studies of 1SS analogs are shown in **Figure 5** in relation to the parent 1SS model (labeled *d* in panel B), insulin lispro (labeled *e* in panel B) and native-state domains N and N* (respectively labeled *f* and *g* in panel B); for clarity, a color code is shown at bottom. Qualitative inspection of the far-UV spectra at 4, 25 and 37°C (**Figures 5A–C**) suggest rank order N* (most structured) > N > insulin *lispro* >> parent 1SS model > 1SS-Ser^B24^ > 1SS-Pro^A16^ > 1SS Pro^B15^ (least structured). These inferences are in accordance with CD deconvolution (**Tables 2A, B**) and two-state thermodynamic modeling (**Table 1** and **Table S1**). Of the 1SS analogs, only the parent model peptide exhibits, to a small extent, a cooperative thermal unfolding curve at low temperatures^3^ (**Figure 5D**) and possibly a cooperative guanidine-denaturation transition with Δ*G*_u_ < 1 kcal/mole (**Figures 5E, F** and **Table S1**).

**Figure 6:**
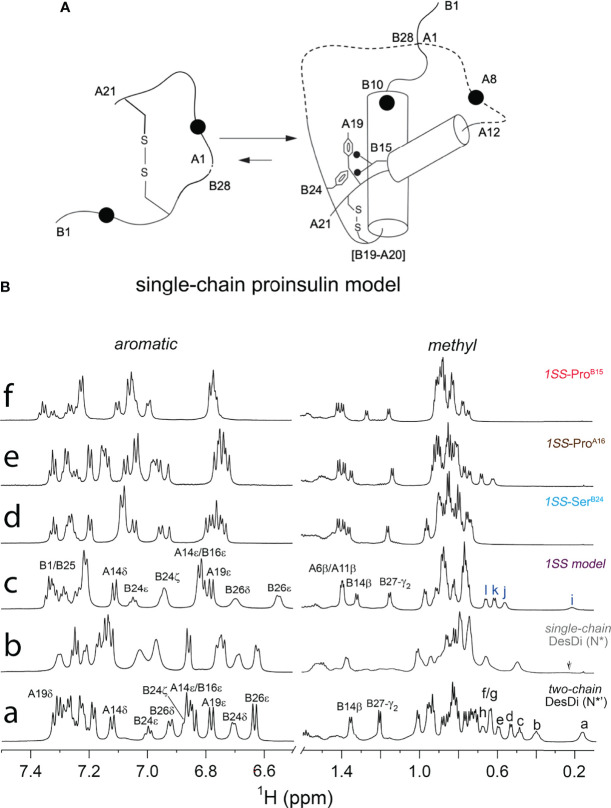
Folding pathway of proinsulin model and NMR spectroscopy. **(A)** Model of proinsulin’s specific folding nucleus based on single-chain 1SS-DesDi analog. Initial pairing of B19-A20 disulfide in combination with the formation of two-helices (B9-B19 and A12-A20) are considered to be central events in the formation of folding nucleus. Dotted lines represent disordered regions. Larger circles represent the Asp^B10^ and Glu^A8^ substitutions. Key residues are highlighted by their sequence position [panel modified from reference (51)] **(B)** Stack plot of 1D ^1^H-NMR spectra of DesDi insulin analogs: aromatic (*left panel*) and methyl (*right panel*) region. (a) two-chain DesDi (N*’). Selected resonance assignments are as indicated in the top of signals. The well-resolved methyl resonances are indicated as a: Leu^B15^ δ_1_-CH_3_; b: Ile^A2^ δ_1_-CH_3_; c: Ile^A10^ δ_1_-CH_3_; d: Leu^B15^ δ_2_-CH_3;_ e: Ile^A2^ γ_2_-CH_3;_ f/g: Ile^A2^ γ_2_-CH_3_ and Leu^B11^ δ_1_-CH_3_; h: Leu^B11^ δ_2_-CH_3_; (b) single-chain DesDi (N*). The arrow indicates broadening signal of Leu^B15^ δ_1_-CH_3_. (c) single-chain DesDi model with one disulfide bond at [B19-A20]. Cys^A6^ and Cys^A11^ were replaced by alanine, and Cys^A7^ and Cys^B7^ were mutated by serine. The 1SS model exhibits a spectral property of native-like insulin as observed in the aromatic region and in the upfield-shifted methyl region (*far right*). Selected resonance assignments are as indicated in the top of signals. The well-resolved methyl resonances are indicated as i: Leu^B15^ δ_1_-CH_3_; j: Leu^B11^ δ_1_-CH_3_; k: Leu^B15^ δ_2_-CH_3_ and l: Leu^B11^ δ_2_-CH_3_. (d) single-chain 1SS-Ser^B24^ analog. (e) single-chain 1SS-Pro^A16^ analog and (f) single-chain 1SS-Pro^B15^ analog. Aromatic and methyl resonances of signature residues for three 1SS variants shifted to downfield, as well as exhibited a reduction in chemical-shift dispersion. Spectra were acquired at pD 7.4 (direct meter reading) at 35°C in D_2_O.

We imagine that the 1SS peptides exist as a conformational equilibrium between a disulfide-tethered random coil (at left in **Figure 6A**) and a collapsed conformation in which nascent α-helical structure occurs in the B domain (residues B9-B19; helix α_1_ in insulin and proinsulin) and A domain (A12-A18; helix α_3_ in insulin and proinsulin). Diffusion-collision of these nascent helices creates a molten proto-core engaging (in the parent model) Leu^B11^, Val^B12^, Leu^B15^, Val^B18^, Phe^B24^, Tyr^B26^, Leu^A16^ and Tyr^A19^ in the neighborhood of internal cystine B19-A20. This scheme envisions that this molten-core functions as proinsulin’s specific folding nucleus (51) and is destabilized by the MIDY mutations but to different extents. Evidence supporting this hypothesis was provided by one-dimensional ^1^H-NMR spectroscopy (**Figure 6B**). Whereas native state analogs (single-chain N* and two-chain N*) exhibit marked chemical-shift dispersion as expected of native globular domains, such dispersion is attenuated among the 1SS analogs in order N* > parent 1SS model > 1SS-Pro^A16^ > 1SS-Pro^B15^. Preservation of chemical-shift dispersion in the proto-core of the parent 1SS peptide is remarkable, as evident by the upfield chemical shifts of aromatic resonances (Phe^B24^, Tyr^B26^ and Tyr^A19^) and aliphatic resonances (Leu^B11^ and Leu^B15^). Qualitative interpretation of the NMR spectrum of Ser^B24^ native state and 1SS analogs was confounded by the absence of the diamagnetic ring-current field of Phe^B24^, a major source of chemical-shift dispersion in native insulin (79). These NMR features are investigated further by two-dimensional heteronuclear NMR in our companion study with focus on ^13^C_α_ and ^1^H_α_ secondary NMR shifts (64).

**Figure 7:**
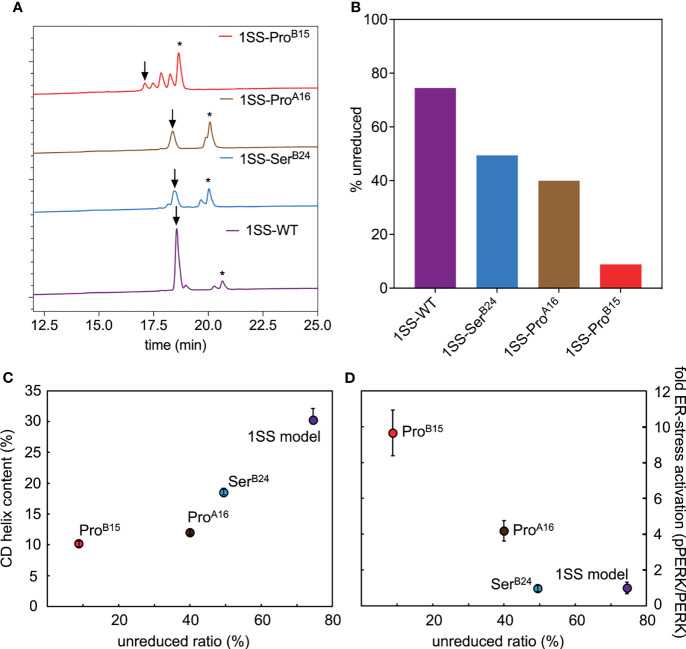
Determination of equilibrium ratios of 1SS analogs in redox buffer containing glutathione reduced (GSH) and glutathione oxidized (GSSH) in 5:1 ratio. **(A)** Reverse-phase HPLC profiles of the equilibrium reaction mixtures after 3 h at 25 °C. Downward arrow indicates the remaining amount of polypeptide (in oxidized state) and asterisk (*) indicates the fully reduced polypeptide. Glutathione adducts were seen intermediate of the oxidized and reduced peaks. **(B)** Bar graph showing percentage remaining unreduced 1SS-polypeptides in the GSH-GSSH redox equilibrium buffer. **(C, D)** Correlations between the stability of 1SS-peptides in redox buffer with other biophysical and biological parameters reflects the same trend as the clinical severity of these mutants. Plots showing the amount of remaining unreduced 1SS-peptides correlates with their CD α-helical content **(C)** and ER-stress levels in a cell-based assay **(D)**.

The relative stabilities of the 1SS proto-cores, although inaccessible to guanidine denaturation studies (above), were instead probed by resistance to reduction at equilibrium in a defined redox buffer (25 mM reduced glutathione and 5 mM oxidized glutathione). Initial solutions contained only the disulfide-constrained peptides and were allowed to come to equilibrium as monitored by serial rp-HPLC chromatograms; representative steady-state chromatograms are shown in **Figure 7A**. Quantitation of the surviving disulfide-constrained elution peaks (arrows in **Figure 7A**) indicates a rank order of redox stability parent 1SS model (most stable) > 1SS-Ser^B24^ > 1SS-Pro^A16^ >> 1SS-Pro^B15^ (least stable; histogram in **Figure 7B**). This trend is in accordance with effects of these mutations on native state DesDi folding yields (**Table 1**) and relative α-helix contents of the 1SS models (**Figure 7C**). Encouraged by this coherence, we speculate that the relative native state folding yields mirror the efficiency of initial closure of cystine B19-A20, in turn dependent on diffusion-collision of the proposed α-helical proto-core.

**Figure 8:**
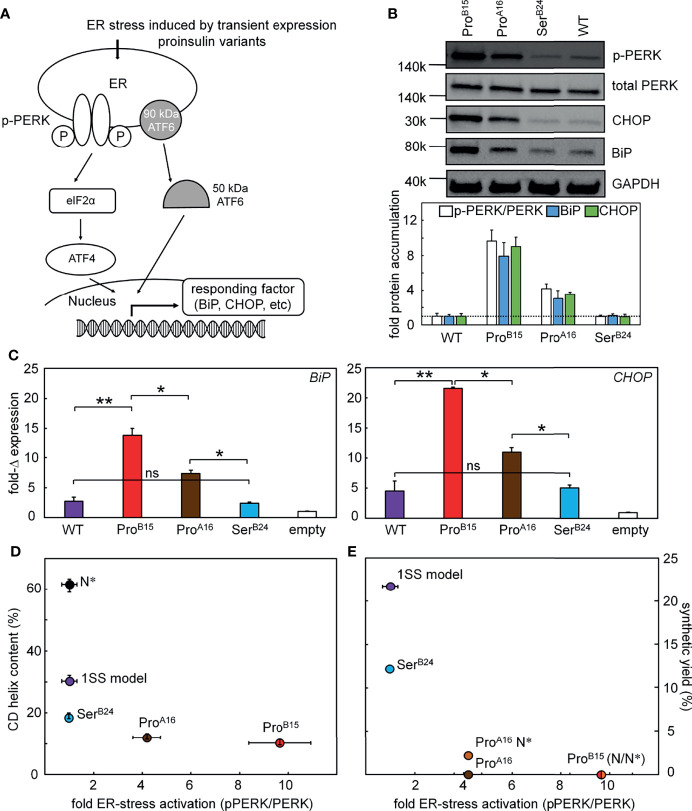
Proinsulin mutants induce ER stress. **(A)** Schematic picture shows that the induced ER stress would be regulated by the expression of different proinsulin variants. Proinsulin variants give rise to accumulation of unfolded protein in the ER resulting in the phosphorylation of PERK and induction of ISR. As common markers, CHOP is a downstream response of the ISR and BiP is a chaperon, which would exhibit increased expression. Western blot was applied to test the protein levels of p-PERK and the accumulations of BiP and CHOP in panel **(B)** The transcription responses of these two ER stress markers were monitored by qPCR assay in panel **(C)**. **(B)** Western-blot assays probing the ER stress markers including p-PERK/PERK, CHOP, and BiP. A total of 3 blots provided ER stress markers: (i) p-PERK alone (due to a specific protocol requirement for antibody), (ii) PERK and GAPDH and (iii) BiP and CHOP. Samples were obtained from the same lysate. WT proinsulin induces low ER stress. Representative gel images (above) shows the WB results and the histogram (bottom) presents the quantification of the WB signals (n=3, biological replicates). **(C)** Real-time qPCR assay probing the transcription responses of *BiP* and *CHOP* genes induced by WT (purple) and variants of proinsulin. Gene markers for ER stress were significantly activated by the expressions of Pro^B15^ (red) and Pro^A16^ (brown) variants but not the Ser^B24^ (blue) proinsulin. Endogenous extent of GAPDH mRNA is applied as internal control to normalize the quantitative analysis of PCR gene expression. Asterisks (*) and (**) indicate p-value < 0.05 and < 0.01. The “ns” indicates p-value > 0.05 (ns - not significant). **(D, E)** Plots show the relationship between cellular ER stress activation and biophysics phenomena. **(D)** The plot shows the relationship between extents of fold of activated ER stress associated with different proinsulin variants (X-axis; using the representative ER stress marker pPERK/PERK ratio) and their percentage of helix contents evaluated by CD (Y-axis). **(E)** Plot showing the result of activated cellular ER stress correlating to the synthetic yield of these analogs in a three-disulfide containing DesDi model. N* indicates Asp^B10^ and Glu^A8^ substitutions that are used to rescue the folding in those cases.

In an effort to connect the above chemical and biophysical properties to cell biology—and ultimately to the pathophysiology of the MPS in patients—we undertook studies of ER stress induced by transient expression of wild-type or mutant proinsulins in a human kidney-derived embryonic cell line readily grown in culture and amenable to transient transfection (HEK 293T cells). Although not related to β-cell lineages, Arvan and colleagues have shown the utility of these cells in studies of proinsulin biosynthesis (**Figure 8A**) (11). ER stress was probed through Western-blot studies of the pPERK/PERK ratio, induction of ER chaperone BiP (a member of the HSP70 family), and ER-stress-responsive transcription factor CHOP (**Figure 8B**). Changes in these markers (relative to the wild-type proinsulin baseline; horizontal dashed line in **Figure 8B**) are shown in **Figures 8C** (BiP on left and CHOP on right). The rank order of ER stress was Pro^B15^ (highest ER stress) > Pro^A16^ > Ser^B24^ > WT > empty vector control. This pattern parallels the sensitivity of the 1SS models to reduction in a defined redox buffer (**Figure 7D**), 1SS α-helix contents (**Figure 8D**) and native-state DesDi folding yields (**Figure 8E**). Such extensive correlations provide evidence that the biophysical and biochemical properties of DesDi 1SS and native-state peptides relate to the pathophysiology of proinsulin biosynthesis in the ER of a human cell.

## Discussion

How proteins fold and misfold define key problems at the intersection of biophysics, cell biology and medicine (80). The mutant proinsulin syndrome highlights the importance of foldability in the process of insulin biosynthesis (81). Mutational impairment of native disulfide pairing in the ER of pancreatic β-cells leads to ER stress, β-cell dysfunction and eventual death (17, 18). This syndrome ordinarily exhibits genetic dominance, implying that misfolding of a variant proinsulin impairs bystander biosynthesis of wild-type proinsulin (19). This monogenic diabetes syndrome thus illuminates structural determinants of specific disulfide pairing (20) and folding efficiency as an implicit evolutionary constraint (23). The present study sought to develop a peptide model of a one-disulfide proinsulin folding intermediate as a general platform for studies of a mechanistic subclass of MIDY mutations: those that impair the nascent conformational search leading to initial pairing of Cys^B19^ and Cys^A20^, an early step in biosynthesis (8, 24). We exploited this platform to investigate three clinical mutations, two with neonatal onset [Pro^B15^ and Pro^A16^ (82, 83)] and one with onset in early adulthood [Ser^B24^ (11, 61, 62)]. The present study extends the use of peptide models of protein-folding intermediates (see **Box 1** (25)] to investigate the molecular pathogenesis of a monogenic syndrome of toxic protein misfolding.

Our peptide model is a 49-residue “mini-proinsulin” based on the DesDi framework as developed by DiMarchi and colleagues to optimize the efficiency of disulfide pairing in an enzyme-cleavable synthetic precursor (50). This framework contains B-chain residues B1-B28 followed by A-chain residues A1-A21. Its Lys^B28^-Gly^A1^ peptide bond (cleavable by protease Lys-C) enables productive folding even of mutant insulins otherwise refractory to classical insulin chain combination (50), presumably by constraining the orientation of A- and B-chain residues to stabilize a specific folding nucleus. We obtained a one-disulfide model through pairwise substitutions of cystines B7-A7 (by serine) and A6-A11 (by alanine). Choice of Ser or Ala was determined by solvent exposure of these disulfide bridges in native insulin (60). Nascent structure in the 1SS model, presumably stabilized by the B28-A1 peptide bond, was further favored by helicogenic substitutions His^B10^→Asp (58) and Thr^A8^→Glu (59). Their additional negative charges were also intended to enhance solubility and mitigate the propensity of partial folds to aggregate *via* exposed nonpolar surfaces. In native state DesDi analogs introduction of these acidic side chains rescues the folding of a Pro^A16^ analog, albeit in small yield. Pro^B15^ blocks folding of DesDi even in the presence of Asp^B10^ and Glu^A8^. Insight into even such deleterious mutations can nonetheless be obtained through studies of corresponding 1SS peptide models.

**Figure 9:**
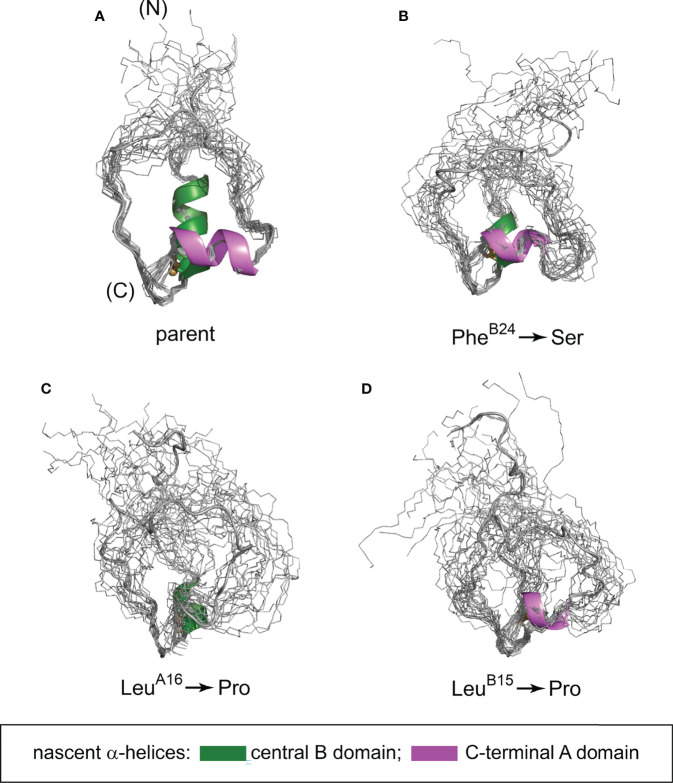
Predicted conformational ensembles of one-disulfide peptide analogs. **(A)** parent model; **(B)** Ser^B24^ variant; **(C)** Pro^A16^ variant and **(D)** Pro^B15^ variant. The ensembles were generated by restrained molecular dynamics using the XPLOR-NIH software (75, 76) and visualized by PyMol (https://sourceforge.net/projects/pymol/). Nascent α-helices in the central segment of the B domain and C-terminal segment of the A domain are respectively shown in green and magenta (box at bottom); predicted helix lengths differ depending on the mutation in accordance with corresponding CD spectra (see **Figure 5** and **Table 2B**).

We have characterized the above three clinical mutations in both native state and 1SS contexts. Physico-chemical properties included oxidative folding yield, nascent structure (including CD-defined α-helix contents), stability to chemical denaturation, and stability to reduction under defined redox conditions. A consistent trend in rank order of perturbations was observed: wild-type > Ser^B24^ >> Pro^A16^ > Pro^B15^ (grossly perturbed). Although CD provides only a low-resolution structural probe, the wealth of prior information (including NMR studies of insulin, proinsulin and homologous growth factors) enables construction of molecular models (**Figure 9**). Intended as working hypotheses, these models highlight the following predicted features:

- *Parent ensemble*. The overall conformation is globular as a partial fold, stabilized by the confluence of a central B-domain α-helix (green ribbon in **Figure 9A**) and C-terminal A-domain α-helix (magenta ribbon). The mini-core contains a native-like cluster of nonpolar and aliphatic side chains (Leu^B11^, Val^B12^, Leu^B15^, Val^B18^ and Leu^A16^) near cystine B19-A20, extended by packing of a nascent C-terminal B-domain β-strand (Phe^B24^ and Tyr^B26^).
- *Ser^B24^ ensemble*. Substitution of Phe^B24^ by Ser would replace aromatic packing against Val^B12^, Leu^B15^ and Cys^B19^ by a small hydrophilic side chain. Our model posits substantial retention of the parent α-helices but with increased flexibility in the N-terminal portion of the B-chain α-helix due to loss of stabilizing Phe^B24^-Val^B12^ and Phe^B24^-Leu^B15^ as well as transmitted destabilization of corresponding long-range Tyr^B26^ contacts.
- *Pro^A16^ ensemble*. Proline has low intrinsic helical propensity (84). Accordingly, substitution of Leu^A16^ by Pro would be expected to destabilize the nascent A-domain α-helix and perturb its packing against the B domain. Our model posits substantial retention of the parent B-domain super-secondary structure but with increased flexibility in these elements and as broadly transmitted in the globule.
- *Pro^B15^ ensemble*. Substitution of Leu^B15^ by Pro would likewise be predicted to destabilize the nascent B-domain α-helix and also introduce multiple long-range perturbations, both to the C-terminal B-domain β-strand (Phe^B24^ and Tyr^B26^) and within the mini-core. Our model posits N-terminal shortening of both B- and A-domain α-helices in accordance with CD spectra (**Figure 5** and **Table 2B**). These segmental and long-range perturbations would be associated with a global enhancement of conformational fluctuations.

Together, our experimental findings and molecular modeling are in accordance with genotype-phenotype relationships as Ser^B24^ (only mildly perturbed in the peptide model) is associated with adult-onset disease (MODY) whereas the more severe Pro substitutions are associated with neonatal onset (PNDM). A direct connection between chemistry and biology was further suggested by correlations between our synthetic and biophysical findings and assays of ER stress induced by the corresponding proinsulin variants in a human cell line. Such coherence among diverse probes validates the present peptide model as a general platform for mechanism-based studies of MIDY mutations mapping near cystine B19-A20. In our companion study in this issue (64), we describe more detailed two-dimensional NMR studies in an effort to deepen the biophysical characterization of this platform. These findings validate major features of the parent model depicted in **Figure 9A**.

Pairing of Cys^B19^ and Cys^A20^ represents only a first step in a complex choreography of disulfide pairing leading to the native state (**Figure 1C**). Indeed, many MIDY mutations map outside of the present folding nucleus as exemplified by substitutions at positions B5, B8 and A4 (18, 20). We envisage that in the future peptide-based strategies can be extended to two-disulfide models that encompass such additional MIDY mutations. Together, reductionist approaches promise to dissect molecular events that underlie aberrant disulfide pairing in a monogenic disease of toxic protein misfolding (17, 18). The evolution of wildtype insulin at the edge of foldability (23) suggests that such studies may inform baseline mechanisms of β-cell ER stress in the natural history of *non-syndromic* Type 2 DM. Structural lessons of the mutant proinsulin syndrome (20) thus promise to uncover a new layer of understanding in deciphering the informational content of insulin sequences (60). This layer, although of only fleeting importance in biosynthesis and hidden once the native state is reached, may nonetheless underlie the phenomenon of β-cell “exhaustion” in Type 2 DM.

Peptide models can facilitate analysis of protein folding by reducing to a minimum the complexity of a globular protein architecture. In favorable cases such simplification can enable critical determinants of folding efficiency to be dissected. We nonetheless caution that such models can be a double-edged sword: the same simplification can lead other structural contributions to foldability (or kinetic obstacles) to be overlooked. In the present case omission of proinsulin’s C domain is likely to introduce offsetting advantages and disadvantages. On the one hand, 1SS DesDi exhibits a surprising richness of structure amenable to high-resolution NMR study (64). On the other hand, insertion of the long and flexible C domain, which impairs the *in vitro* refolding efficiency of single-chain insulin analogs, may allow a broader set of native and non-native disulfide bridges to be formed (8), some as off-pathway kinetic traps (43, 85, 86). Further, the present model pertains to only a subset of clinical mutations; other models may be required to investigate mutations distant from cystine B19-A20. These caveats notwithstanding, the complex biophysical chemistry of disulfide pairing in proinsulin, considered in its entirety, poses a foundational problem in protein science, central to the pathogenesis of β-cell dysfunction as a pandemic disease of civilization.

## Data Availability Statement

The original contributions presented in the study are included in the article/**Supplementary Material**. Further inquiries can be directed to the corresponding authors.

## Ethics Statement

The animal study was reviewed and approved by Institutional Animal Care and Use Committee.

## Author Contributions

Chemical peptide syntheses were performed by BD, AZ, MJ, and RD. CD studies were performed by BD, MG, and NP. ER stress assays were conducted by Y-SC. NMR studies were performed and interpreted by YY, and MW. Figures were prepared by BD, MG, AZ, Y-SC, and MW. Supplemental rat studies were overseen by NP and FI-B. The Supplement was prepared by BD, AZ, YY, and MW. All authors contributed to editing the manuscript with first draft prepared by BD, MG, and MW. Overall experimental design and oversight were provided by MW. All authors contributed to the article and approved the submitted version.

## Funding

This work was supported in part by grants to MW from the National Institutes of Health (R01 DK040949 and R01 DK069764). AZ were supported in part by Calibrium, LLC. NP was supported in part by the American Diabetes Association (grant no. Grants 7-13-IN-31 and 1-08-RA-149). MG was a Pre-doctoral Fellow of the National Institutes of Health (Medical Scientist Training Program 5T32GM007250-38 and Fellowship 1F30DK104618-01).

## Conflict of Interest

This study received partial funding from Calibrium, LLC. The funder was not involved in the study design, collection, analysis, interpretation of data, the writing of this article or the decision to submit it for publication. All authors declare no other competing interests.

## Publisher’s Note

All claims expressed in this article are solely those of the authors and do not necessarily represent those of their affiliated organizations, or those of the publisher, the editors and the reviewers. Any product that may be evaluated in this article, or claim that may be made by its manufacturer, is not guaranteed or endorsed by the publisher.

## Footnotes

1 Normalized CD spectra are unaffected by changes in peptide concentration in the 20-90 μM range.

2 CD -based estimates of secondary structure content are in good agreement with published values based on SELCON-3 deconvolution (77).

3 For single-chain N and N* parent domains, their limited temperature dependence of in the range 4-40°C correlates with enhanced folding efficiency and increased thermodynamic stability.

## Abbreviations

MIDY, mutant *INS*-induced diabetes of the young; MODY, maturity-onset diabetes of the young; MPS, mutant proinsulin syndrome; PNDM, permanent neonatal diabetes mellitus; DesDi, single-chain insulin analog insulin containing Lys^B28^ and lacking residues B29 and B30; N, DesDi insulin template with all three native disulfide bonds; N*, DesDi insulin control with solubility enhancements; [B19-A20], DesDi variant based on N* retaining only the B19-A20 disulfide linkage; ER, endoplasmic reticulum; SCI, single-chain insulin; CD, circular dichroism; NMR, nuclear magnetic resonance; rp-HPLC, reverse-phase high-performance liquid chromatography; LC-MS, liquid chromatography-mass spectrometry; MALDI-TOF, matrix-assisted laser desorption ionization - time of flight; and IGF, insulin-like growth factor. Insulin residues are denoted by residue type (in standard three-letter code) followed by the chain and position (e.g., Phe^B24^ designates a phenylalanine at the 24^th^ position of the B chain).
