# Supplementary material for: Peptide Model of the Mutant Proinsulin Syndrome. I. Design and Clinical Correlation
Source: Front Endocrinol (Lausanne). 2022 Mar 1;13:821069. doi: 10.3389/fendo.2022.821069 (PMC8922534; doi:10.3389/fendo.2022.821069)
Supplement: Supplementary file 1 [file DataSheet_1.pdf]

Supplemental Information

for

Peptide Model of the Mutant Proinsulin Syndrome. I. Design and Clinical Correlation

Balamurugan Dhayalan[†,*], Michael D. Glidden[†], Alexander N. Zaykov[†], Yen-Shan Chen, Yanwu Yang, Nelson B. Phillips, Faramarz Ismail-Beigi, Mark A. Jarosinski, Richard D. DiMarchi, & Michael A. Weiss[*]

Table of Contents

Table of Contents 1

Purpose of Supplement 2

Supplemental Discussion on the pH conditions affecting protein folding. 3

Table S1 4

Figure S1 5

Figure S2 6

Figure S3 7

Figure S4 7

Figure S5 8

Figure S6 8

Figure S7 9

Figure S8 9

Figure S9 10

Figure S10 10

Figure S11 11

Figure S12 11

Figure S13 12

Figure S14 12

Figure S15 13

Figure S16 14

References 15

Purpose of Supplement

This Supplement contains one table and 16 figures. In Supplemental Table S1 are given reverse-phase HPLC (rp-HPLC) retention times for synthetic insulin analogs (containing three native disulfide bridges) and corresponding 49-residue polypeptide models (designated "1SS"), predicted and observed molecular masses as determined by mass spectrometry, and estimates of free energies of unfolding (ΔG_u) as inferred from CD-monitored chemical denaturation studies. Supplemental **Figures S1** and **S2** provide rp-HPLC chromatograms documenting oxidative folding reactions (or their failure) pertaining to native three-disulfide insulin analogs. These analogs are in the framework of either single-chain *des*-[B29, B30]-Lys^{B28}-human insulin ("DesDi"; (1)) or a stabilized analog, single-chain Asp^{B10}-Glu^{A8}-DesDi. The native states of single-chain DesDi and single-chain [Asp^{B10}, Glu^{A8}]-DesDi are respectively denoted N and N*. The B10 and A8 substitutions in N* enhance segmental helical propensity and overall thermodynamic stability.

Supplemental **Figures S3-S15** provide LC-MS profiles. These document the synthesis and purification of parent single-chain N and N* analogs (**Figure S3** and **S4**), Ser^{B24} and Pro^{A16} single-chain analogs (**Figure S5** and **S6**), enzymatic conversion of N and N* to two-chain analogs (**Figure S7** and **S8**), enzymatic conversion of Ser^{B24} and Pro^{A16} single-chain precursors to two-chain analogs (**Figure S9** and **S10**), corresponding 1SS analogs (**Figures S11-S14**) and enzymatic conversion of WT-1SS to two-chain (**Figure S15**).

Although this article focuses on folding efficiency (via a novel one-disulfide single-chain peptide model of a key proinsulin folding intermediate) rather than classical structure-activity relationships in the mature two-chain hormone, activities of corresponding two-chain analogs were evaluated in vivo. Enzymatic cleavage of the single-chain synthetic precursors is required for functional assessment because the peptide bond between Lys^{B28} and Gly^{A1} in the precursor otherwise blocks bioactivity (as in other mini-proinsulins; (1, 2)). **Figure S16** thus provides functional data in male Lewis rats (mean body mass ~300 g) rendered diabetic by streptozotocin. Time-courses of blood-glucose concentrations are plotted either as absolute values (in mg/dL; panels **A-D** on *left*) and relative to starting level of glycemia (**E-H** on *right*).

The rat studies are in general accordance with expectations based on extent of structural perturbations shown in the main text. Pro^{A16} inactivates two-chain *des*-[B29, B30]-Lys^{B28}-DesDi (**Figure S16**, panels **A** and **E**) in accordance with its overall destabilization and loss of organized structure. Additional data confirm previous reports that Ser^{B24} impairs the activity of a two-chain analog (panels B and F; (3)); near-full activity is regained on administration of a supra-therapeutic dose (panels C and G). Such partial loss of activity is in accord with perturbation of a conserved receptor contact as the aromatic side chain of Phe^{B24} engages Site 1 of the receptor ectodomain ((4)). Finally, the one-disulfide 49-residue polypeptide, when split into two chains, is also without detectable activity (panels D and H), presumably due to flexibility of the potential receptor-binding surface in a molten partial fold. Theoretical considerations suggest that binding of the two-chain 1SS model would be markedly weakened by the free-energy cost of folding on the receptor. Because the two-chain parent peptide was inactive, studies of 1SS analogs (Ser^{B24}, Pro^{A16} or Pro^{B15}) were not performed. We note in passing that a homologous 1SS recombinant model of IGF-I was previously found to exhibit very low, but detectable activity at high hormone concentrations ((5)).

Supplemental Discussion on the pH conditions affecting protein folding

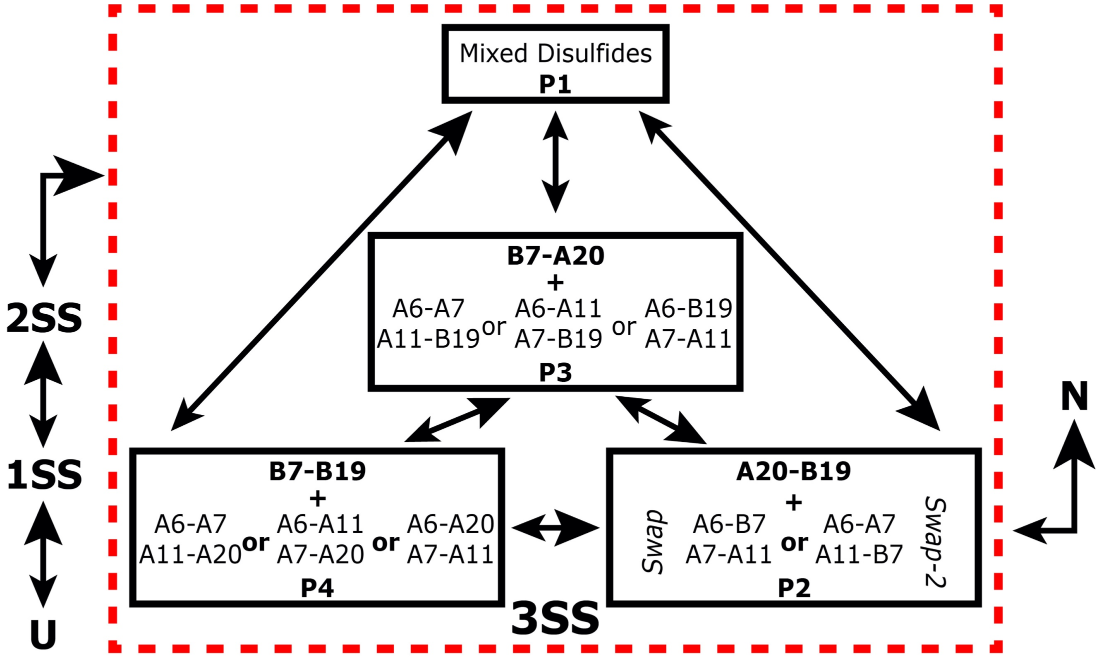

Scheme S1. Folding of human proinsulin at pH 10.5 lead to formation of multiple disulfide isomers (6). Solid boxes encase 3SS intermediates that have been isolated in refolding studies (named P1, P2, P3, and P4) whereas bold labeled disulfide pairs are linkages confirmed by mass spectrometry. P2 exists in two non-native disulfide arrangements that correspond to those of insulin-*swap* and insulin-*swap2* (as defined by Hua et al. (7, 8)).

The purpose of this Supplemental Discussion is to highlight confounding differences in the proinsulin folding mechanisms under pH-10.5 conditions relative to neutral-pH conditions. **Scheme 1** is based on the refolding assays of human proinsulin at pH 10.5. At these conditions (which lead to deprotonation of cysteine and hence chemical activation of the thiolate) four different intermediates were obtained with three disulfide bonds (rectangle boxes in **Scheme 1**) (6). However, refolding studies of the shortened single-chain porcine insulin precursor (PIP) at neutral pH provided evidence that folding proceeds through the formation of one- and two-sulfide linked intermediates before the native state is reached (9). This study also suggested that P2 (consisting of the canonical B19-A20 disulfide linkage) is a favored intermediate that must be formed before the native state is reached (10). Complementary evidence of folding intermediate was observed in studies of IGF-1: refolding at neutral pH (7.5) leads to two isomers, one with native pairing (IGF-1) and another non-native pairing (IGF-*swap*), but *both products proceed through a common one-disulfide-linked intermediate* (Cys18-Cys61, homologous to cystine B19-A20 in proinsulin) (11).

Refolding conditions can also alter effects of MIDY mutations in proinsulin. Indeed, pH 10.5 conditions have been found to improve—and even completely rescue—otherwise non-foldable clinical mutants at physiological pH (as exemplified by L-Ser^{B8}-proinsulin (12)). Remarkably, the in-vitro folding yield of wild-type proinsulin is increased from 20% to 90% by raising the pH to 10.5. This is why the latter pH is employed in large-scale manufacturing of proinsulin by Eli Lilly and Co., i.e., to optimize yield.

TABLE S1. Molecular properties of peptide models and insulin analogs[a]

polypeptide	HPLC retention time (min)	Molar Mass (Da) theoretical / actual	ΔG_u (kcal/mol)[b]
A)			
Insulin *lispro*	17.12	5807.5 / 5806.7	3.0 ± 0.1
Wild-type Insulin	17.28	5807.5 / 5806.2	3.1 ± 0.1
N	14.77	5591.4 / 5590.4	>4[c]
N*	15.20	5597.3 / 5596.2	7.6 ± 0.4[c]
Ser^{B24} single-chain (N)	15.73	5531.3 / 5530.3	3.4 ± 0.1[c]
Pro^{A16} single-chain (N*)	20.43	5581.3 / 5580.7	0.9 ± 0.1
two-chain DesDi (N)	16.96	5609.4 / 5608.3	2.8 ± 0.1
two-chain DesDi (N*)	20.96	5615.3 / 5614.3	4.9 ± 0.1
Ser^{B24} two-chain (N)	16.00	5549.3 / 5548.2	1.6 ± 0.1
Pro^{A16} two-chain (N*)	20.21	5599.2 / 5598.1	N.D.[d]
B)			
1SS-WT	23.57	5505.0 / 5504.2	0.9 ± 0.2
1SS-Ser^{B24}	22.03	5445.0 / 5444.0	ND[d]
1SS-Pro^{B15}	18.77	5489.0 / 5488.4	ND[d]
1SS-Pro^{A16}	21.71	5489.0 / 5488.1	ND[d]

[a]Analytical LC-MS retention times (column 2) and theoretical and experimentally measured molar masses (column 3) are given for all DesDi species not labeled with ^{13}C.

[b]ΔG_u values provided were obtained from curve fitting of CD-guanidine titrations to a two-state unfolding transition model as described in Materials and Methods.

[c]CD-guanidine titrations performed at 37 °C for N and 50 °C N* (see **Figure 4** in main text) yielded partially folded peptides at the maximum guanidine concentration. Fitting of the parent N* titration was in accord with a two-state transition (R^2=0.9994), but analysis of the parent N titration curve could only place a lower bound on thermodynamic stability of 4 kcal/mol.

[d]ND, *not determined* due to lack of a well-defined pre-transition ellipticity baseline corresponding to the folded state; the denaturation data are not in accordance with a cooperative two-state transition and suggest instead a molten globule.

Analytical data

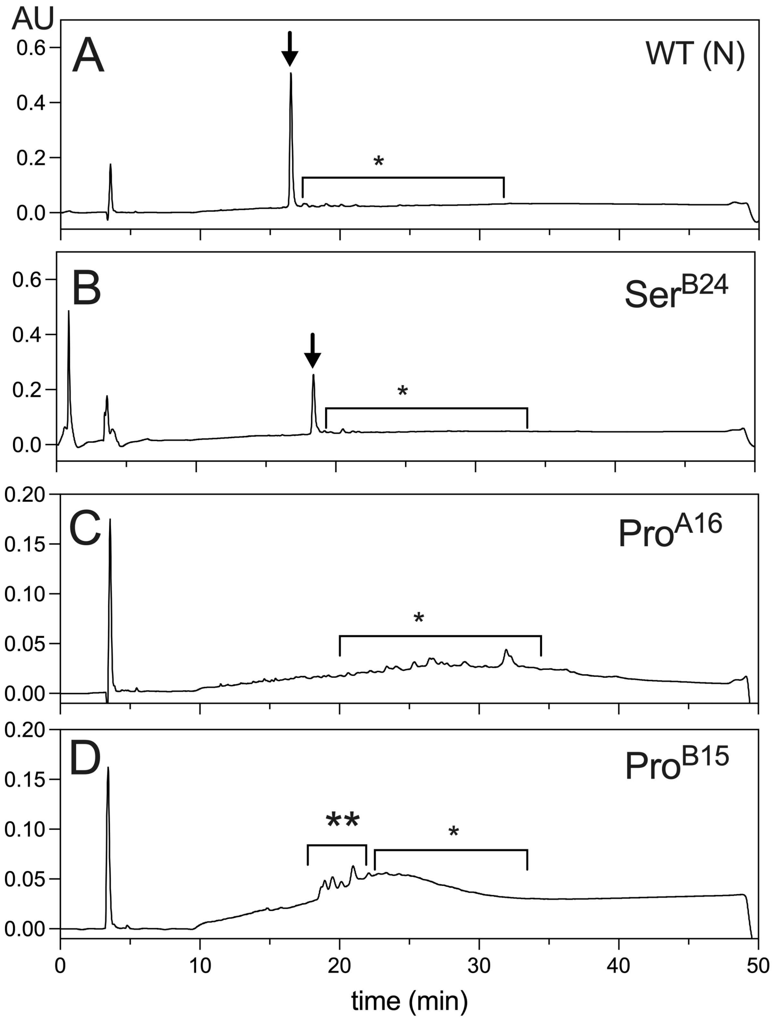

FIGURE S1 | Folding profiles (rp-HPLC traces monitored UV absorbance at 215 nm) of DesDi analogs (N). Correctly folded DesDi is indicated by downward arrow; * represents misfolded state; ** represents disulfide isomers.

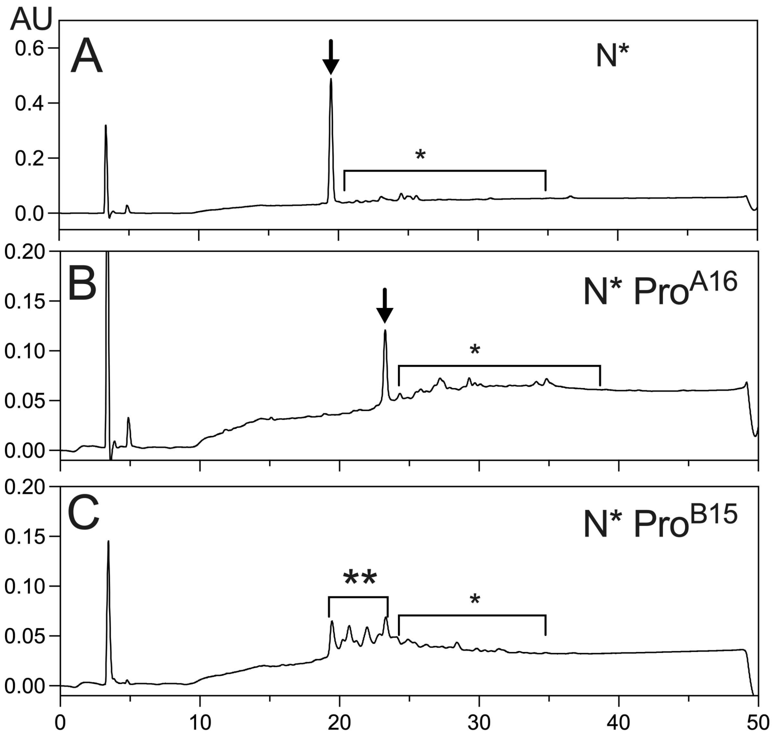

FIGURE S2 | Folding profiles (rp-HPLC traces monitored by UV absorbance at 215 nm) of N* DesDi analogs. N* has Asp^{B10} and Glu^{A8} substitutions for the wild-type residues. Correctly folded DesDi is indicated by downward arrow; * represents misfolded state; ** represents disulfide isomers.

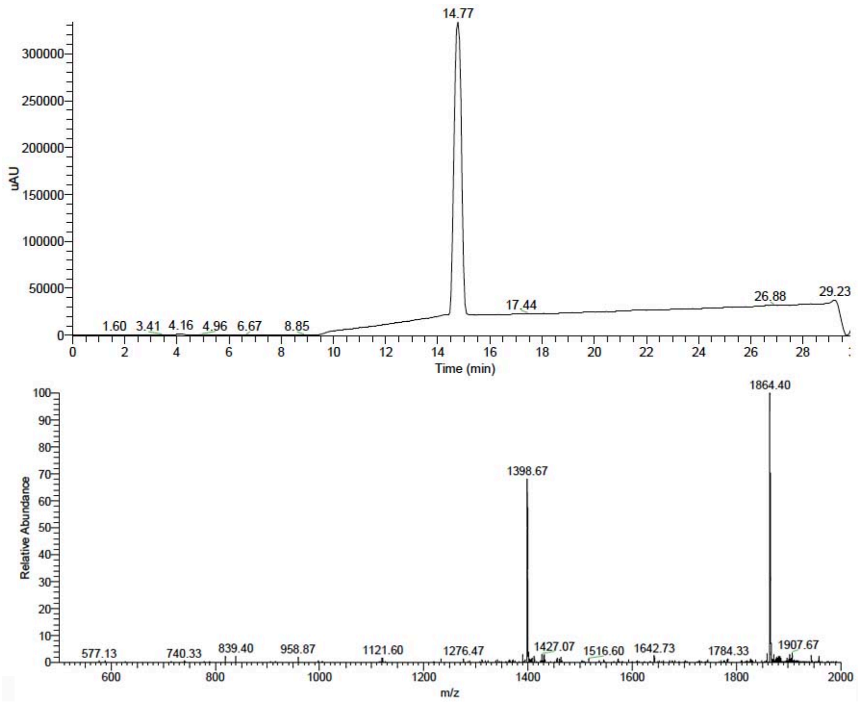

FIGURE S3 | LC-MS analysis of wild-type DesDi (N). HPLC chromatogram (monitored by UV absorbance at 215 nm) is shown at the top panel. ESI-MS spectra are shown in bottom panel.

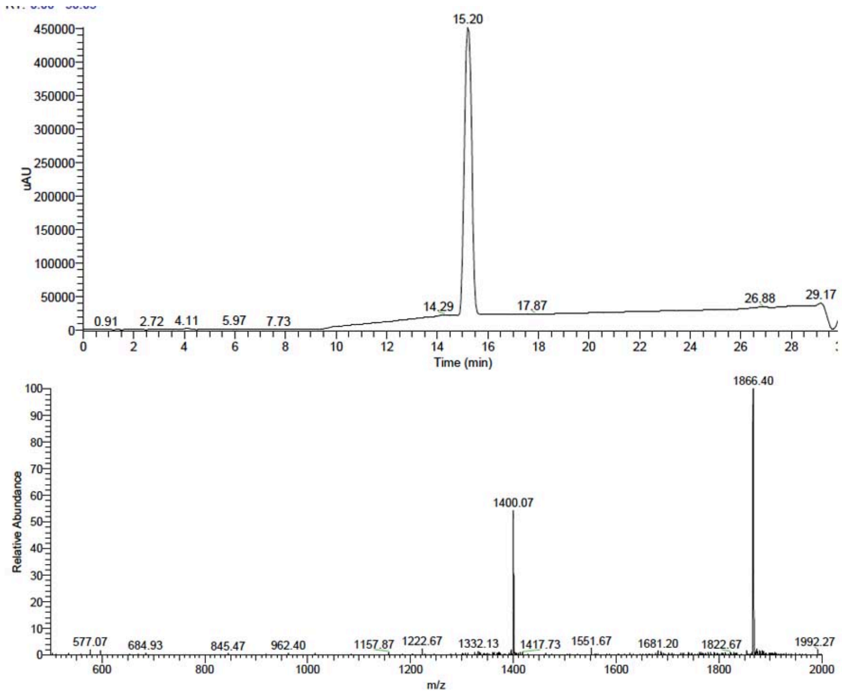

FIGURE S4 | LC-MS analysis of [Glu^{A8}, Asp^{B10}] DesDi (N*). HPLC chromatogram (monitored by UV absorbance at 215 nm) is shown at the top panel. ESI-MS spectra are shown in bottom panel.

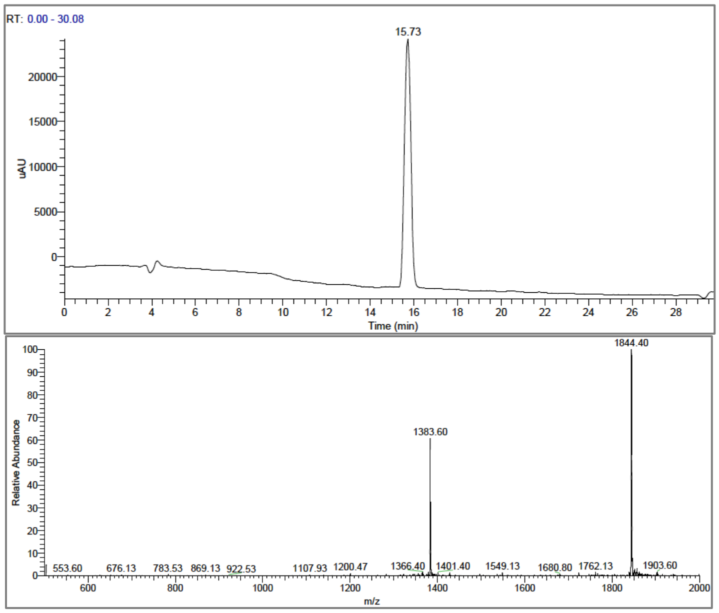

FIGURE S5 | LC-MS analysis of Ser^{B24} DesDi single-chain analog. HPLC chromatogram (monitored by UV absorbance at 215 nm) is shown at the top panel. ESI-MS spectra are shown in bottom panel.

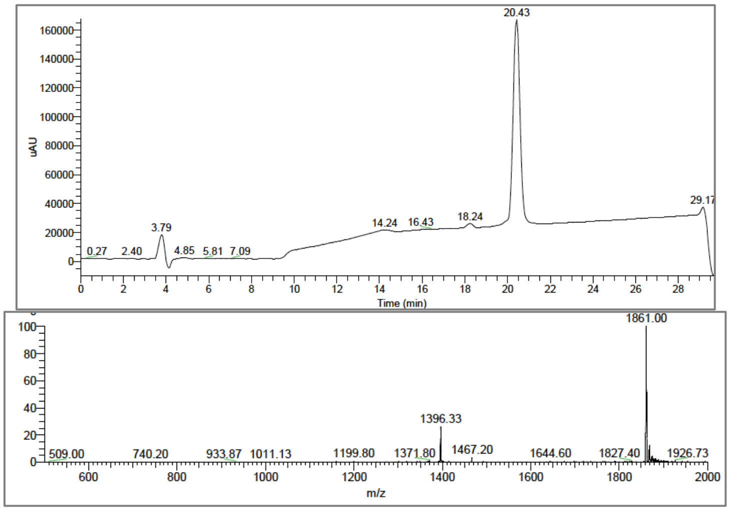

FIGURE S6 | LC-MS analysis of Pro^{A16} (N*) DesDi single-chain analog. HPLC chromatogram (monitored by UV absorbance at 215 nm) is shown at the top panel. ESI-MS spectra are shown in bottom panel.

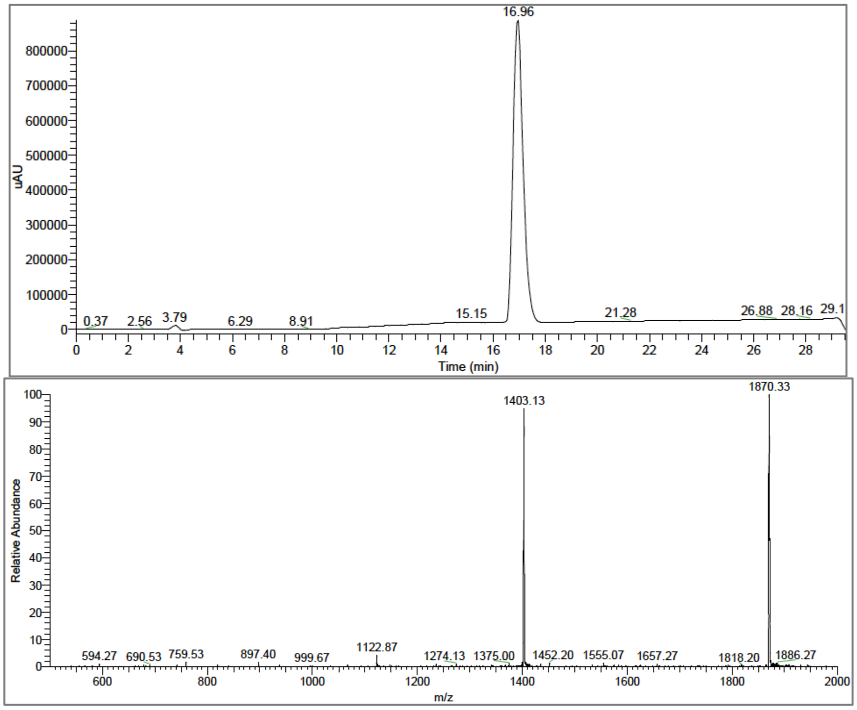

FIGURE S7 | LC-MS analysis of wild-type DesDi two-chain analog. HPLC chromatogram (monitored by UV absorbance at 215 nm) is shown at the top panel. ESI-MS spectra are shown in bottom panel.

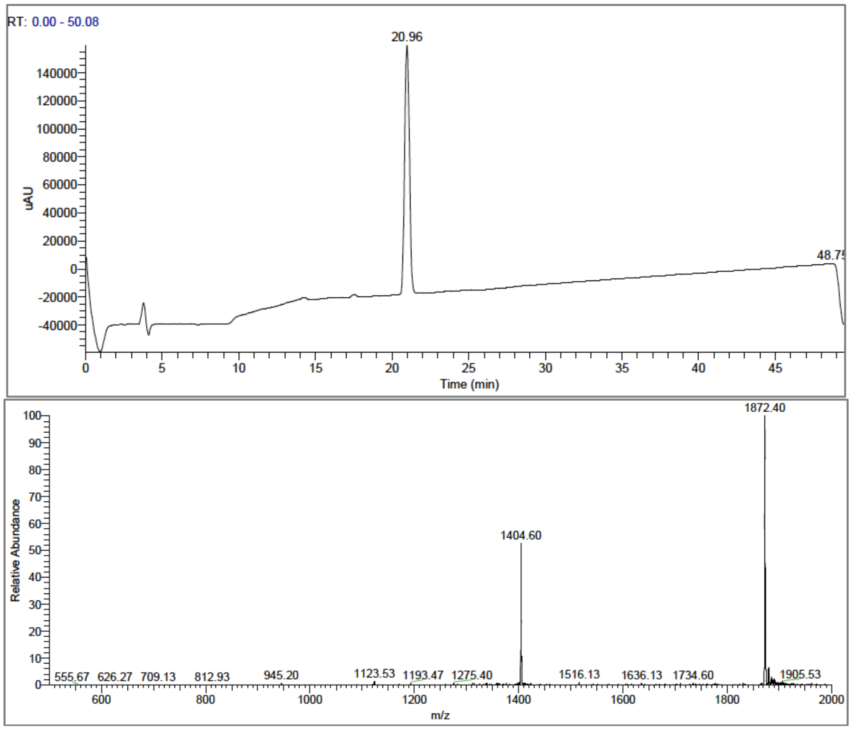

FIGURE S8 | LC-MS analysis of [Glu^{A8}, Asp^{B10}] DesDi (N*) two-chain analog. HPLC chromatogram (monitored by UV absorbance at 215 nm) is shown at the top panel. ESI-MS spectra are shown in bottom panel.

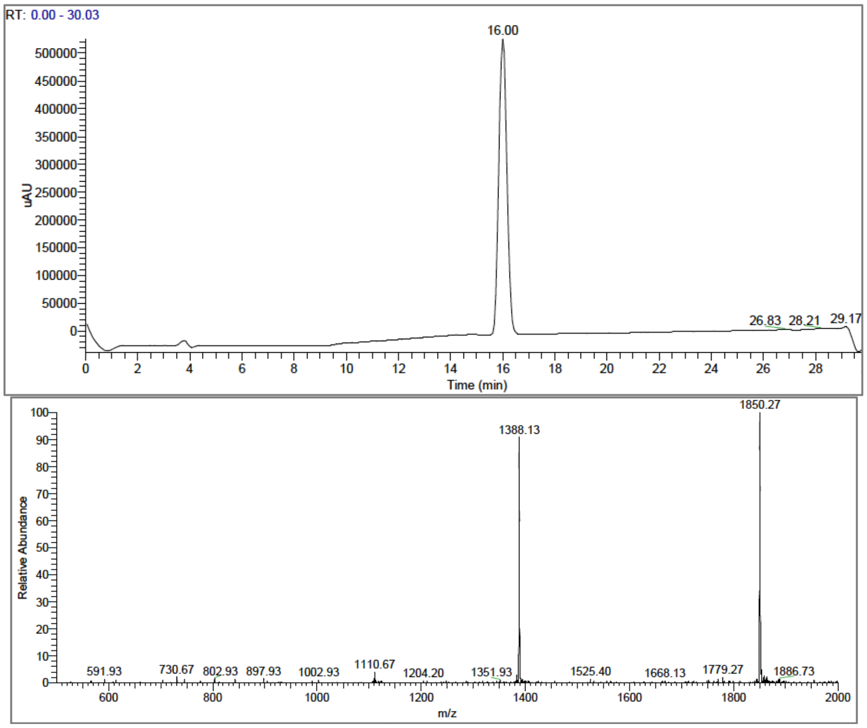

FIGURE S9 | LC-MS analysis of Ser^{B24} DesDi two-chain analog. HPLC chromatogram (monitored by UV absorbance at 215 nm) is shown at the top panel. ESI-MS spectra are shown in bottom panel.

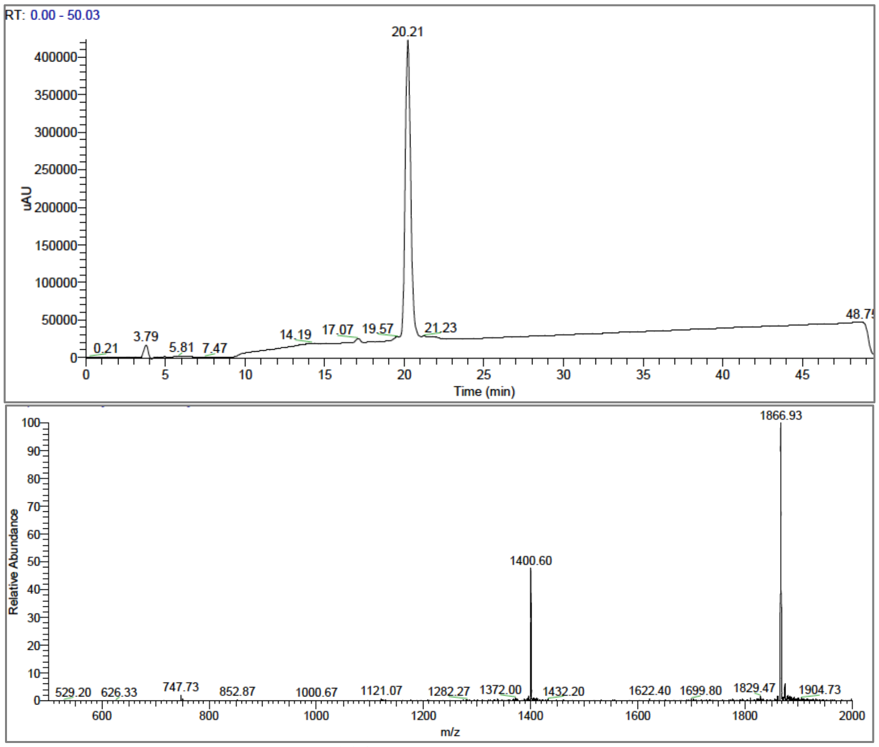

FIGURE S10 | LC-MS analysis of Pro^{A16} DesDi (N*) two-chain analog. HPLC chromatogram (monitored by UV absorbance at 215 nm) is shown at the top panel. ESI-MS spectra are shown in bottom panel.

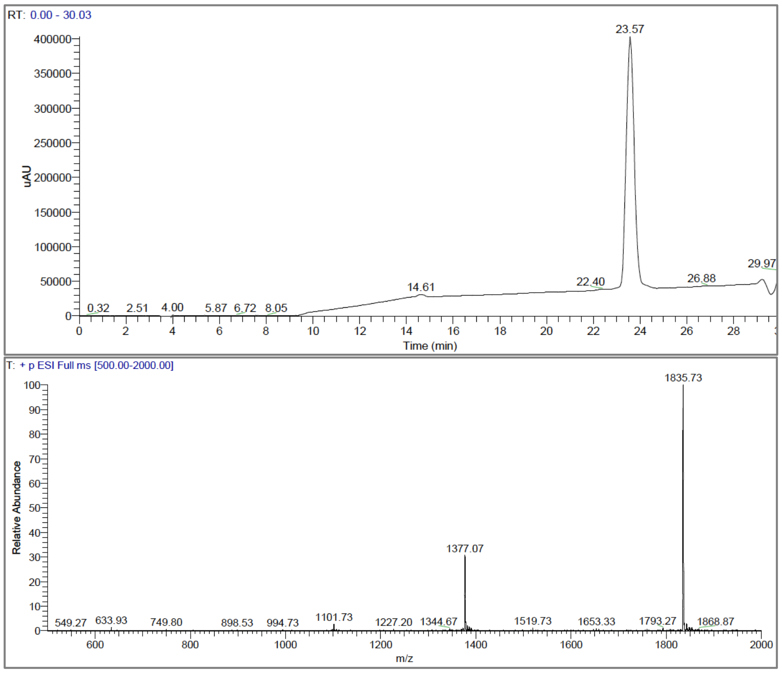

FIGURE S11 | LC-MS analysis of 1SS-WT DesDi analog. HPLC chromatogram (monitored by UV absorbance at 215 nm) is shown at the top panel. ESI-MS spectra are shown in bottom panel.

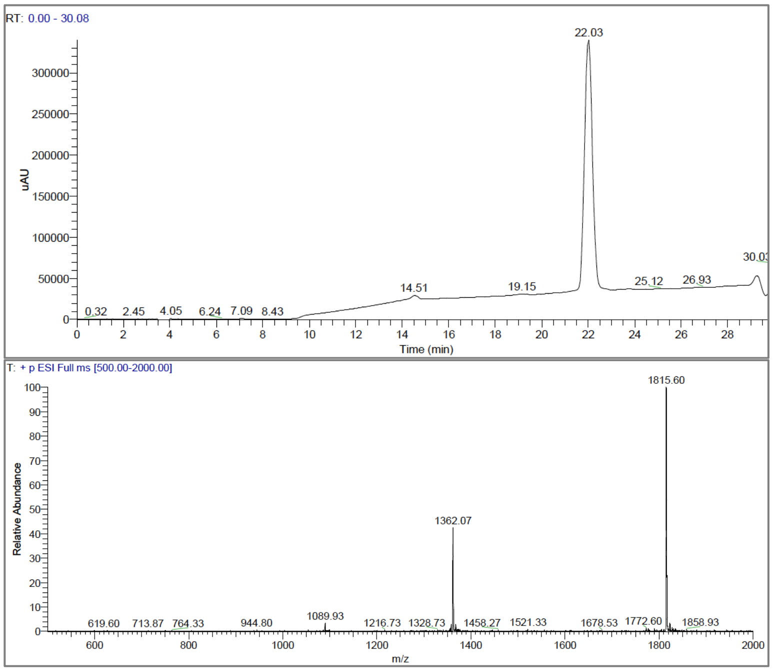

FIGURE S12 | LC-MS analysis of 1SS-Ser^{B24} DesDi analog. HPLC chromatogram (monitored by UV absorbance at 215 nm) is shown at the top panel. ESI-MS spectra are shown in bottom panel.

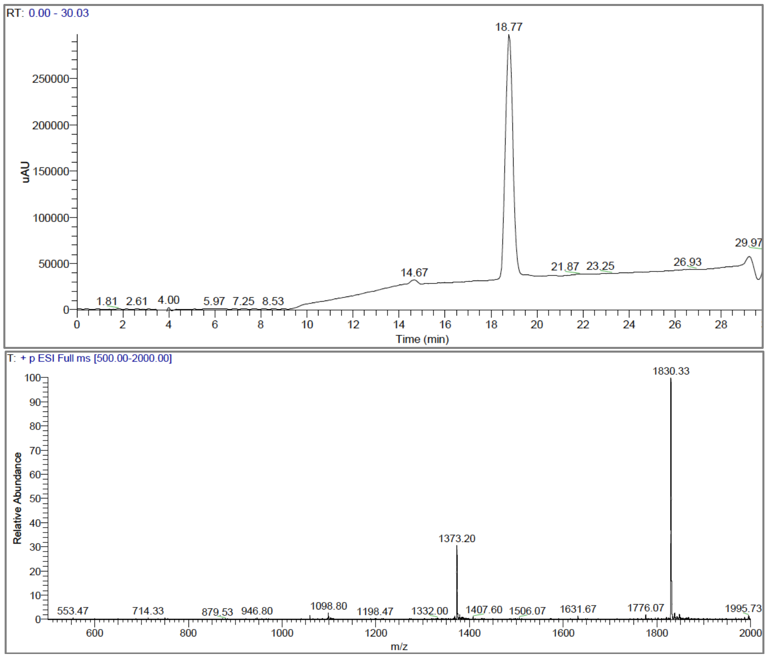

FIGURE S13 | LC-MS analysis of 1SS-Pro^{B15} DesDi analog. HPLC chromatogram (monitored by UV absorbance at 215 nm) is shown at the top panel. ESI-MS spectra are shown in bottom panel.

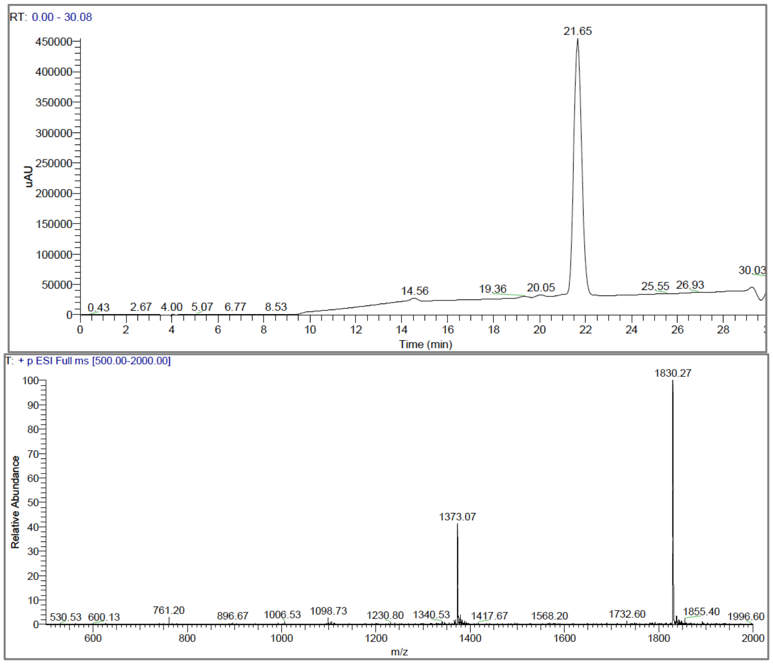

FIGURE S14 | LC-MS analysis of 1SS-Pro^{A16} DesDi analog. HPLC chromatogram (monitored by UV absorbance at 215 nm) is shown at the top panel. ESI-MS spectra are shown in bottom panel.

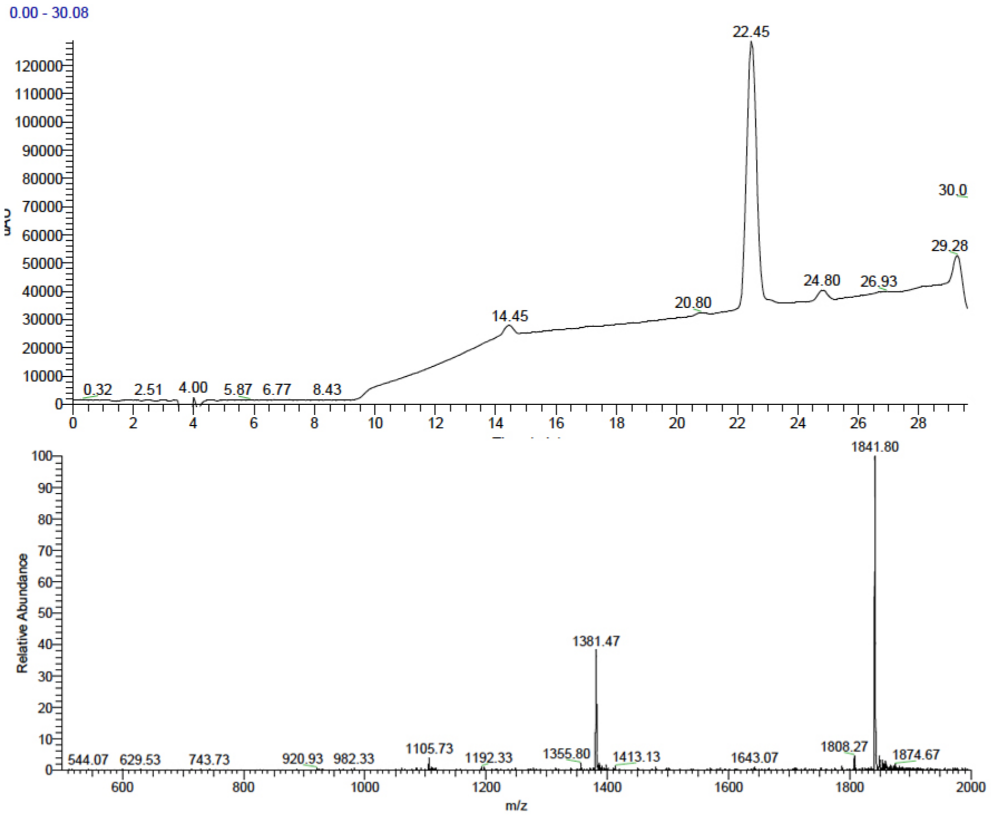

FIGURE S15 | LC-MS analysis of 1SS-WT two-chain analog. HPLC chromatogram (monitored by UV absorbance at 215 nm) is shown at the top panel. ESI-MS spectra are shown in bottom panel.

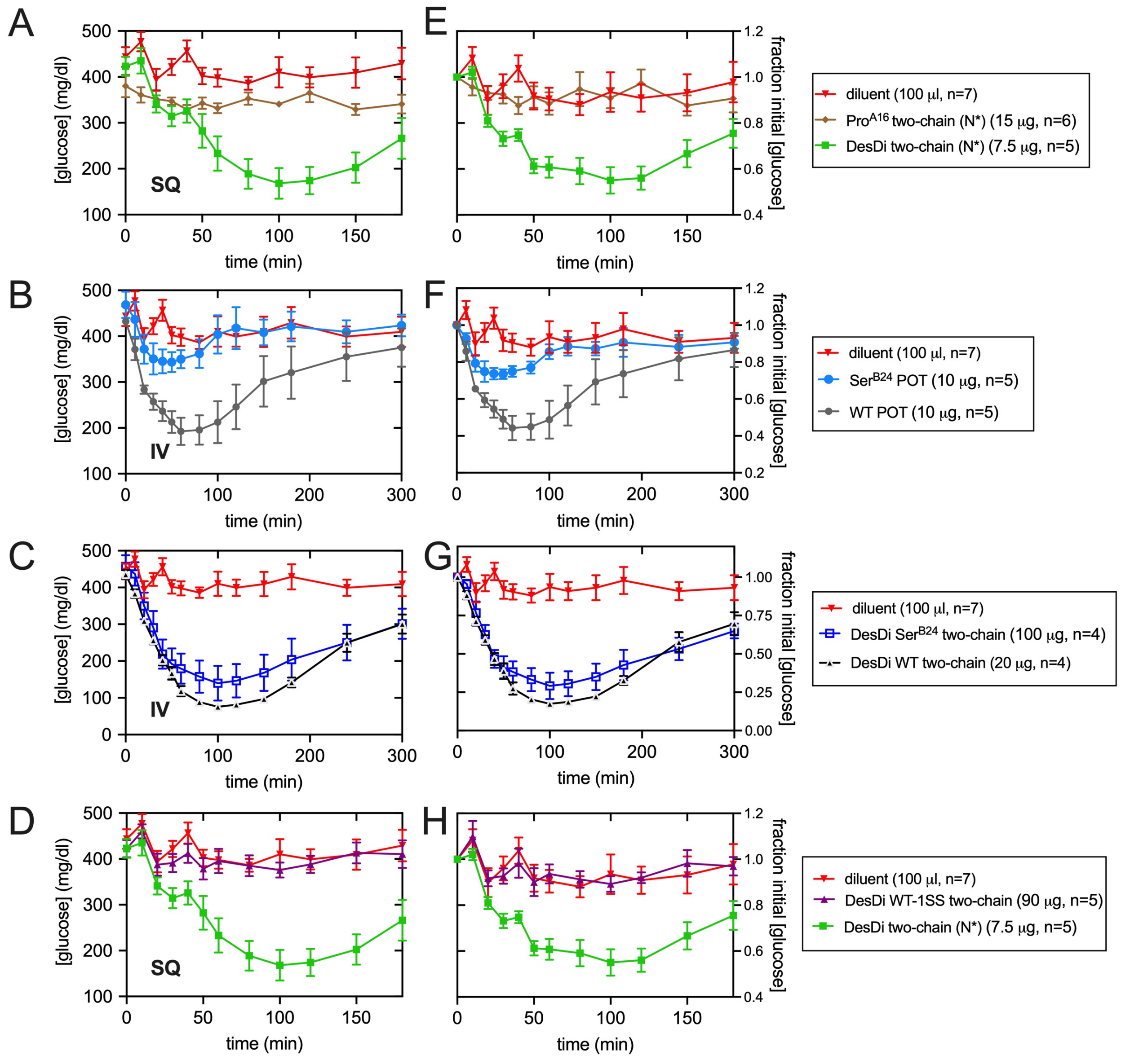

FIGURE S16 | Rat studies of DesDi two-chain insulin analogs. (**A**) time course of [blood glucose] following Subcutaneous injection of Pro^{A16} DesDi (N*) two-chain analog and controls showing negligible activity for this analog (p value < 0.05). N* represent Asp^{B10}, Glu^{A8} substitutions in place of wild-type residues. (**B**) time course of [blood glucose] following intravenous injection of Ser^{B24}-POT at a nominal dose showing lower potency compared to the control (POT denotes Orn^{B29}-insulin; i.e., Pro^{B28}-Orn^{B29}-Thr^{B30}) (p value < 0.05). (**C**) time course of [blood glucose] following intravenous injection of Ser^{B24}-POT at higher dose shows significant activity (p value is 0.1654). (**D**) time course of [blood glucose] following Subcutaneous injection of two-chain 1SS-WT showing no activity even at higher doses (p value < 0.05). (**E**-**H**), normalized blood glucose profiles for the data in panels **A**-**D**, respectively. Sample names, doses and number of rats employed are presented on the right-hand panel. Standard errors of mean are given by vertical error bars. Statistical significance was determined using ANOVA; a p value of <0.05 was considered significant whereas p values just above this threshold may suggest a trend whose validation would require larger studies.

References

1. Zaykov AN, Mayer JP, Gelfanov VM, DiMarchi RD. Chemical synthesis of insulin analogs through a novel precursor. *ACS Chem Biol*. 2014;9(3):683-91.
2. Markussen J, Jørgensen KH, Sørensen AR, Thim L. Single chain des-(B30) insulin: Intramolecular crosslinking of insulin by trypsin catalyzed transpeptidation. *Int J Pept Protein Res*. 1985;26(1):70-7.
3. Shoelson S, Haneda M, Blix P, Nanjo A, Sanke T, Inouye K, et al. Three mutant insulins in man. *Nature*. 1983;302:540-3.
4. Menting JG, Yang Y, Chan SJ, Phillips NB, Smith BJ, Whittaker J, et al. Protective hinge in insulin opens to enable its receptor engagement. *Proc Natl Acad Sci U S A*. 2014;111(33):E3395-E404.
5. Narhi LO, Hua QX, Arakawa T, Fox GM, Tsai L, Rosenfeld R, et al. Role of native disulfide bonds in the structure and activity of insulin-like growth factor 1: genetic models of protein-folding intermediates. *Biochemistry*. 1993;32:5214-21.
6. Qiao Z-S, Min C-Y, Hua Q-X, Weiss MA, Feng Y-M. In vitro refolding of human proinsulin kinetic intermediates, putative disulfide-forming pathway, folding initiation site, and potential role of C-peptide in folding process. *J Biol Chem*. 2003;278(20):17800-9.
7. Hua QX, Gozani SN, Chance RE, Hoffmann JA, Frank BH, Weiss MA. Structure of a protein in a kinetic trap. *Nat Struct Biol*. 1995;2:129-38.
8. Hua QX, Jia W, Frank BH, Phillips NB, Weiss MA. A protein caught in a kinetic trap: structures and stabilities of insulin disulfide isomers. *Biochemistry*. 2002;41:14700-15.
9. Qiao ZS, Guo ZY, Feng YM. Putative disulfide-forming pathway of porcine insulin precursor during its refolding in vitro. *Biochemistry*. 2001;40:2662-8.
10. Yan H, Guo ZY, Gong XW, Xi D, Feng YM. A peptide model of insulin folding intermediate with one disulfide. *Protein Sci*. 2003;12:768-75.
11. Miller JA, Narhi LO, Hua QX, Rosenfeld R, Arakawa T, Rohde M, et al. Oxidative refolding of insulin-like growth factor 1 yields two products of similar thermodynamic stability: a bifurcating protein-folding pathway. *Biochemistry*. 1993;32:5203-13.
12. Avital-Shmilovici M, Whittaker J, Weiss MA, Kent SB. Deciphering a molecular mechanism of neonatal diabetes mellitus by the chemical synthesis of a protein diastereomer,[D-AlaB8] human proinsulin. *J Biol Chem*. 2014;289(34):23683-92.
